# Phenotypic Analysis of Edible Mushroom Fruiting Bodies in Monocular RGB Images: A Problem-Oriented Critical Review

**DOI:** 10.3390/jof12090704

**Published:** 2026-09-21

**Authors:** Wei Zhao, Xin Tian, Lu Yuan, Yinglong Wang, Quan Wei, Hua Yin, Ziwei Song

**Affiliations:** 1 School of Software, Jiangxi Agricultural University, Nanchang 330045, China; zhaowei2096@163.com (W.Z.); lu_yuan8990@126.com (L.Y.); 2 College of Computer and Information Engineering, Jiangxi Agricultural University, Nanchang 330045, China; wangyl@jxau.edu.cn (X.T.); 17770275218@163.com (Y.W.); 3 Ministry of Education Key Laboratory of Crop Physiology, Ecology and Genetic Breeding, Jiangxi Agricultural University, Nanchang 330045, China; quanw8781@163.com

**Keywords:** edible mushroom fruiting bodies, monocular RGB imaging, phenotypic analysis, morphological measurement, spatial information estimation

## Abstract

Monocular RGB imaging offers a low-cost, flexible approach to morphological measurement, quality assessment, growth monitoring, and production automation for edible mushroom fruiting bodies. However, the relationships among visible phenotypes, visual methods, measurement reliability, and production requirements remain insufficiently integrated. This problem-oriented review synthesizes 76 core studies published between 2016 and the final search date in 2026 through a framework linking visible phenotypes, visual tasks, key bottlenecks, and production applications. It covers individual localization and separation, structural measurement, spatial estimation, quality and species recognition, temporal analysis, and production deployment. The reviewed studies reveal a transition from static two-dimensional detection and counting toward instance-level morphological measurement, three-dimensional parameter estimation, and spatiotemporal growth modeling, extending phenotyping from visible appearance description to spatial trait estimation and growth prediction. Quantitative results also highlight the importance of evaluation conditions: for example, MSH-YOLOv8 achieved an ${\mathrm{AP}}_{50}$ of 98.49% on the Fungi dataset, whereas ${\mathrm{AP}}_{50:95}$ and small-object AP were 75.29% and 39.73%, respectively, indicating that high ${\mathrm{AP}}_{50}$ alone does not adequately characterize detection performance under stricter localization criteria or for small targets. These study-specific results cannot be directly extrapolated to commercial production environments. Severe occlusion, projection errors, inconsistent phenotype definitions, and temporal instability continue to constrain measurement reliability. Moreover, reliable performance without extensive retraining following changes in strains, substrates, or lighting systems remains insufficiently demonstrated. Future research should prioritize standardized multi-task and temporal datasets, unified phenotype definitions, uncertainty evaluation, occlusion-robust spatial and temporal modeling, and closed-loop production validation. Commercial scaling of low-cost monocular RGB imaging in protected mushroom cultivation depends on translating its affordability into reliable performance under severe occlusion and across production conditions, thereby enabling accessible automation for small and medium-scale producers.

## 1. Introduction

Edible mushrooms constitute an important component of protected agriculture and high-value agricultural production, with substantial significance for food supply, nutritional health, and the resource utilization of agricultural waste [1,2,3]. With the development of factory-based cultivation, precise environmental regulation, and intelligent equipment, edible mushroom production is gradually shifting from traditional experience-driven management toward data-driven and intelligent decision-making [4,5]. In this process, the low cost, continuous, and objective acquisition of growth status, appearance quality, and maturity information has become an important foundation for yield estimation, quality grading, harvest decision-making, and automated management [6]. As a key link between biological growth processes and production management decisions, phenotypic analysis is playing an increasingly important role in intelligent edible mushroom production.

Phenotypic analysis of edible mushrooms can involve mycelia, spores, and fruiting bodies at different developmental stages, which differ substantially in imaging scale, phenotypic indicators, and application objectives. Mycelial phenotyping usually focuses on colony expansion, texture variation, contamination detection, and culture-state evaluation, whereas spore phenotyping relies more heavily on microscopic imaging and micromorphological analysis [7]. In contrast, fruiting bodies are the direct objects of commodity formation, maturity assessment, quality evaluation, and harvesting operations. Their visible characteristics, including number, size, morphology, color, texture, cap opening degree, and spatial distribution, directly affect commodity grade, yield estimation, harvest timing, and production scheduling [8,9]. Therefore, this review focuses on phenotypic analysis of edible mushroom fruiting bodies.

Among various phenotypic acquisition approaches, monocular RGB images offer the advantages of low equipment cost, simple acquisition procedures, flexible deployment, and intuitive data representation. Image acquisition can be achieved using ordinary smartphones, network cameras, or industrial cameras. Compared with depth cameras, spectral imaging systems, and multi-sensor systems, monocular RGB imaging requires lower hardware investment, less complex system calibration, reduced maintenance, and simpler data processing [10,11]. These characteristics are particularly relevant to continuous monitoring in cultivation houses, where mushroom growth requires high relative humidity [12]. Under such conditions, humidification and local temperature differences may lead to near-saturation conditions and condensation, making optical-window protection and condensation control important deployment considerations. Airborne fungi and spore dispersal have also been documented in mushroom cultivation facilities [13], making contamination of exposed optical surfaces a further maintenance consideration. Engineering guidance for Zivid 3D cameras explicitly notes that condensation or dust on lenses and optical windows may degrade point-cloud quality and recommends humidity control and regular cleaning [14]. For systems that depend on optical projection and reception or sensor registration, the effects of protective measures on measurement stability and maintenance requirements should therefore be evaluated for the specific equipment. Monocular RGB cameras likewise require protection against moisture and condensation, together with optical cleaning. Their practical advantage lies in a simpler hardware configuration and calibration procedure. Imaging choices should consequently account for information requirements, environmental suitability, and ongoing maintenance demands.

Image analysis techniques have long been used for mushroom morphological measurement and variety discrimination, indicating that two-dimensional information such as length, width, and shape descriptors has certain value for phenotypic representation [15]. In recent years, computer vision and machine learning methods have further advanced tasks such as edible mushroom recognition [16], localization, grading, quality evaluation [17], and automated harvesting [18]. For small and medium-scale edible mushroom producers, low cost and ease of deployment are often more practically important than obtaining the most complete information. In particular, in scenarios such as mushroom house inspection, grading lines, harvest assistance, and long-term image recording, monocular RGB images can provide continuous appearance information at a relatively low technical threshold, thereby serving as a fundamental data source for fruiting body detection, counting, measurement, quality recognition, and maturity assessment.

Existing reviews have examined intelligent perception and phenotypic analysis of edible mushrooms from different perspectives. In 2022, our research group published what was, to the best of our knowledge, the first systematic review of computer vision and machine learning applications in the edible mushroom industry [19]. That study covered mushroom recognition, localization, harvesting, grading, and quality evaluation, and compared the characteristics and limitations of two-dimensional vision, three-dimensional vision, and machine learning methods in edible mushroom production. Over the past four years, the field has advanced rapidly, expanding toward instance segmentation, fine-grained morphological measurement, temporal growth monitoring, lightweight deployment, and production-oriented visual systems.

Zhao et al. [20] subsequently summarized advances in deep learning-based edible mushroom phenotyping and intelligent environmental control, including two-dimensional morphological analysis, three-dimensional reconstruction, spectral feature fusion, temporal growth modeling, environment–phenotype coupling, and closed-loop control. Their work reflects the broader transition from isolated visual recognition tasks toward multimodal perception, dynamic modeling, and intelligent regulation. Similarly, Jacob et al. [21] reviewed artificial intelligence applications in edible mushroom cultivation, breeding, and classification, covering intelligent environmental control, disease detection, yield prediction, germplasm characterization, automated classification, and grading. This synthesis further demonstrates the expansion of mushroom intelligence toward multimodal perception, intelligent breeding, and production-system optimization.

Nevertheless, these reviews address relatively broad research domains. The capability boundaries, failure mechanisms, and production applicability of monocular RGB imaging remain insufficiently examined in relation to the visible phenotypes of fruiting bodies. Although this imaging modality is inexpensive and easy to deploy, the absence of direct depth measurements introduces uncertainty in depth and scale that may propagate and accumulate across phenotype extraction, state assessment, and production execution. For example, when two-dimensional projected dimensions are used as approximations of physical dimensions, biases associated with imaging distance, viewpoint, or fruiting-body orientation may affect size grading and size-based maturity assessment. Inaccurate estimates of spatial position or occluded structures may, in turn, affect harvesting localization, target selection, and path planning. High detection or classification performance on a particular dataset therefore does not establish the reliability of the resulting phenotype measurements or automated decisions. Existing reviews provide insufficient integration of information loss, phenotype error, and downstream decision consequences within a framework that examines whether these errors have been identified, quantified, and validated against production outcomes. A systematic problem-oriented synthesis is therefore still needed to determine whether fruiting bodies can be consistently detected, densely distributed individuals can be accurately separated, structural traits can be reliably measured, quality and maturity can be robustly assessed, and the resulting visual phenotypes can effectively support harvesting, grading, yield estimation, and other production decisions.

**Figure 1:**
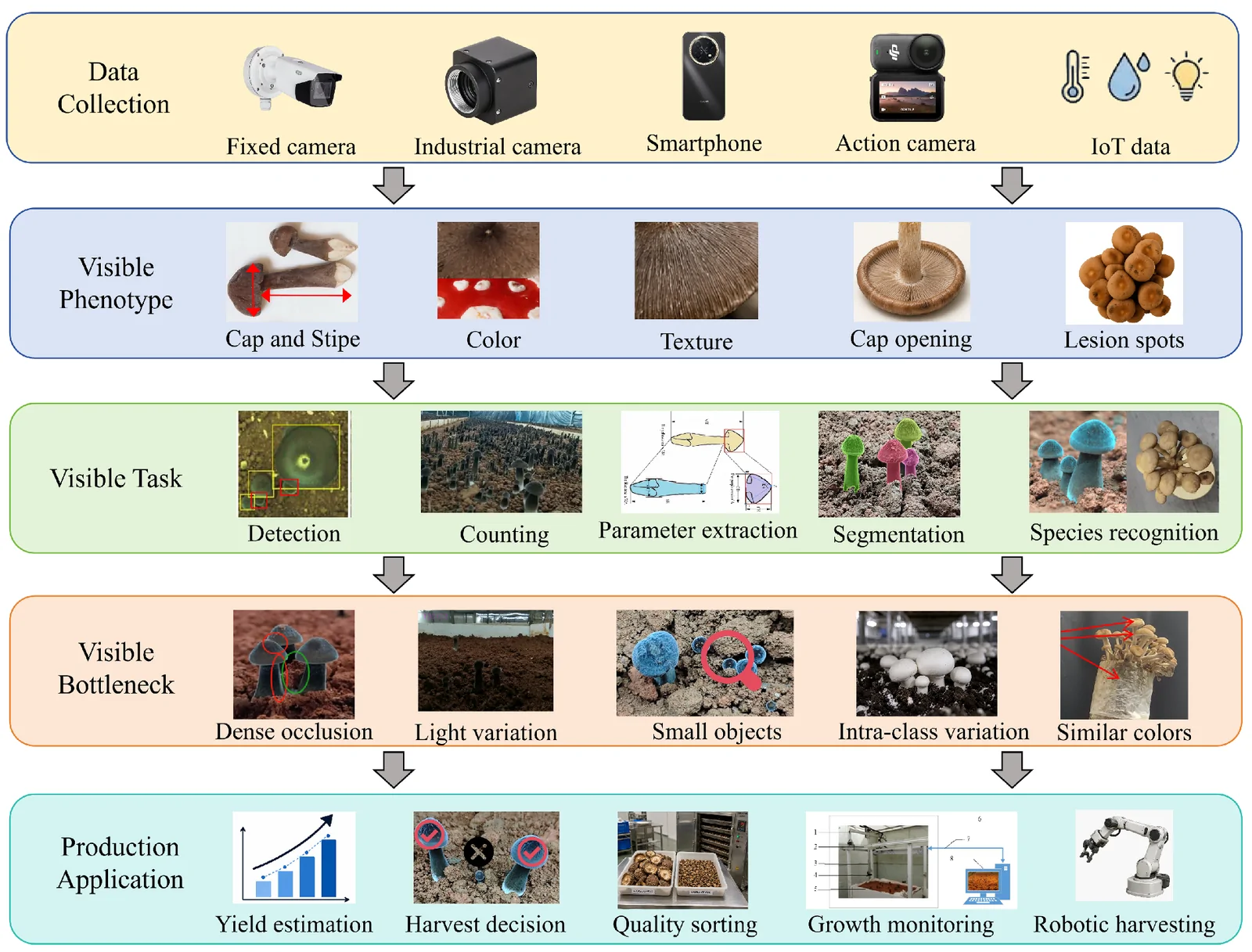
Analytical framework of this review.

To address this need, the present study focuses on visible phenotypic analysis of edible mushroom fruiting bodies using monocular RGB images. Rather than organizing the literature primarily by algorithm category or sensor type, the analysis follows the visible phenotypes–visual tasks–key bottlenecks–production applications framework shown in Figure 1. The framework serves both to organize the literature and to audit the evidence for technical reliability. It connects phenotypes, methods, and applications while examining whether the evidence is sufficient to support practical deployment: whether target traits are directly observable, which geometric assumptions or learned priors underpin inferred outputs, under which conditions methods may fail, and whether phenotype errors have been linked to production outcomes through validation. The observable phenotypic information is first organized into three levels: morphological and structural traits, appearance quality, and growth and maturity status. Correspondences are then established between visual tasks, including detection, segmentation, measurement, classification, tracking, and prediction, and production-related indicators such as cap diameter, projected area, roundness, quality grade, maturity status, and harvest window. Finally, existing methods are critically examined in terms of target visibility, individual separability, structural measurability, state discriminability, process traceability, and result usability, with emphasis on their methodological evolution, applicability boundaries, and remaining gaps. This approach distinguishes evidence of algorithmic performance under specific test conditions from evidence of reliability in industrial environments, and identifies the additional validation needed to translate visual analysis into sustained production use.

## 2. Methods

### 2.1. Literature Search, Study Selection, and Data Extraction

**Figure 2:**
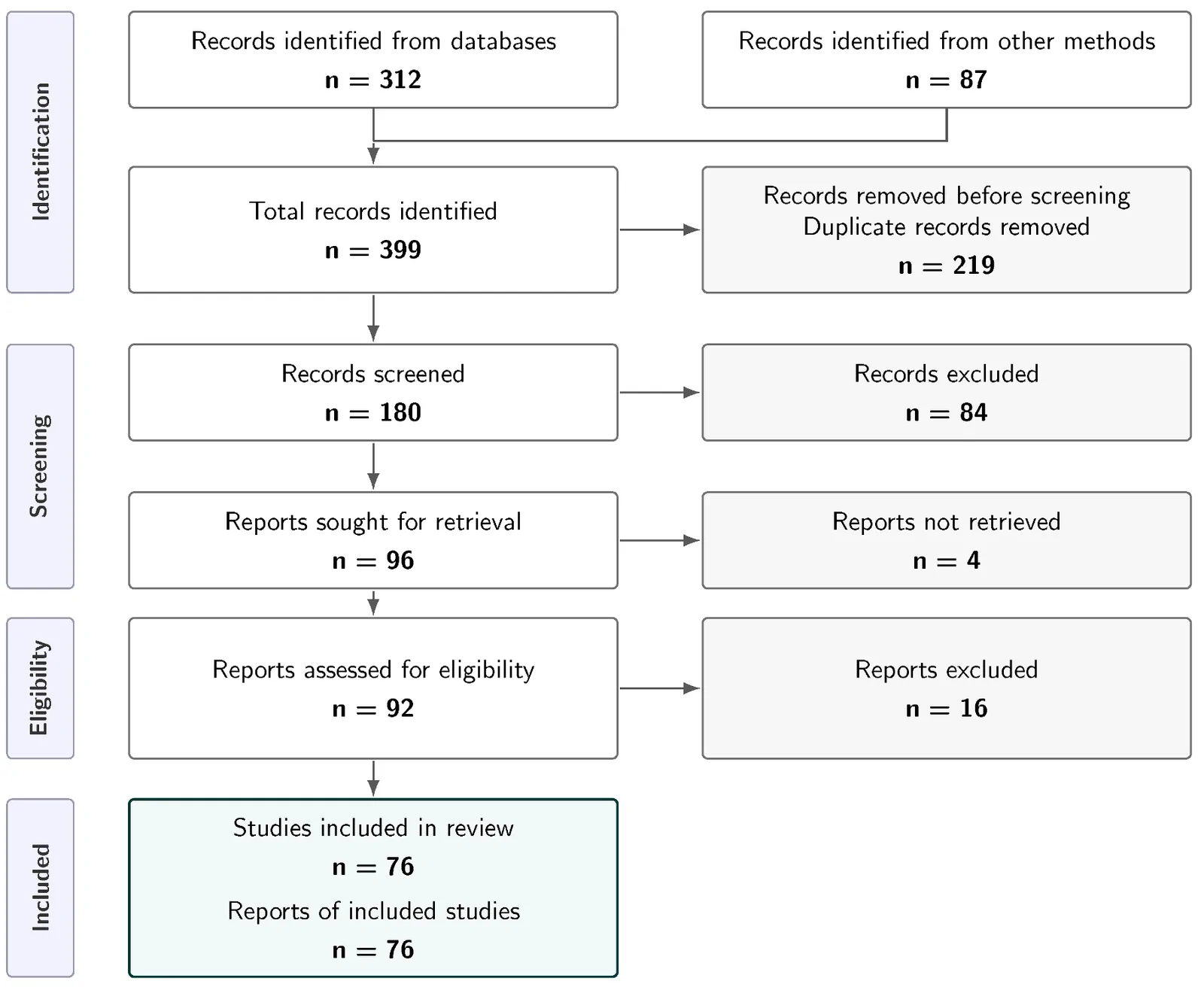
Literature search and selection flow diagram prepared with reference to the relevant PRISMA 2020 reporting items. The diagram presents record counts, full-text retrieval, and final inclusion across four phases: identification, screening, eligibility assessment, and inclusion. A total of 76 core studies, corresponding to 76 reports, were included.

This study is a problem-oriented critical review. To improve the transparency and traceability of literature selection, search sources, inclusion and exclusion criteria, and the numbers of records and reports at each stage are reported with reference to the items relevant to literature searching and study selection in the Preferred Reporting Items for Systematic Reviews and Meta-Analyses (PRISMA) 2020 statement [22]. The study selection process is presented in four phases: identification, screening, eligibility assessment, and inclusion, as shown in Figure 2.

Studies published from 2016 to the date of the final literature search in 2026 were searched to identify research on RGB image-based phenotypic analysis of edible mushroom fruiting bodies and related production applications. English-language studies were retrieved mainly from Google Scholar, Web of Science, Scopus, ScienceDirect, IEEE Xplore, SpringerLink, and PubMed, while Chinese-language studies were searched through the China National Knowledge Infrastructure and Wanfang Data databases. Search terms covered four dimensions: research object, imaging modality, visual task, and application objective. Representative terms included mushroom, fruiting body, RGB image, monocular vision, phenotyping, detection, segmentation, morphological measurement, quality grading, species classification, maturity assessment, growth monitoring, depth prediction, three-dimensional reconstruction, and harvesting robot, together with equivalent Chinese terms and related combinations. Reference lists of key studies and reviews were also examined.

A two-level literature set was established. Core studies were used for descriptive and thematic analyses, whereas contextual references were retained only for background discussion and methodological comparison. Core studies were required to be original research published within the specified period, to use standard RGB or two-dimensional visible-light images as the primary input, to focus on edible mushroom fruiting bodies or directly related image-based production scenes, and to address at least one phenotyping or production-related visual task with sufficient methodological and experimental information. The final inclusion count reported in the flow diagram refers to the core study set; references used solely for background discussion and methodological comparison are not included in this count.

Studies focusing exclusively on mycelia, spores, molecular or chemical analysis, nutritional quality, non-image-based environmental control, or structured non-image attributes were excluded. Studies based primarily on RGB-D, structured light, dedicated depth sensors, directly acquired point clouds, spectral or thermal imaging, or multi-sensor fusion were also excluded. Monocular RGB methods that computationally estimated depth or spatial information were retained, together with one representative multi-view RGB reconstruction study as a boundary extension.

During identification, database searches yielded 312 records, and an additional 87 records were identified by examining the reference lists of key studies and reviews and using connected papers to identify related literature, resulting in 399 records in total. Before screening, 219 duplicate records were removed, leaving 180 unique records. Screening of titles, abstracts, and keywords excluded 84 records that did not meet the review scope or inclusion criteria, and the full texts of the remaining 96 reports were sought for retrieval. Four reports could not be retrieved, leaving 92 reports for full-text eligibility assessment. Based on the inclusion and exclusion criteria described above, 16 reports were excluded following full-text assessment. The final set comprised 76 core studies, corresponding to 76 reports.

For the 76 included core studies, information on publication year, research object, acquisition method, visual task, technical approach, extracted traits, evaluation metrics, application objective, and major limitations was recorded.

The studies were assigned to six mutually exclusive categories: small-object localization and individual separation; structural segmentation, occlusion recovery, and morphological measurement; RGB-based three-dimensional reconstruction and spatial estimation; appearance-based quality assessment and species identification; temporal growth analysis and maturity assessment; and production-oriented applications. Studies involving multiple tasks were classified according to their primary objective and methodological contribution.

The illustrative mushroom photographs in this review were acquired by the authors using a Canon EOS 90D (Canon Inc., Tokyo, Japan), a Redmi K20 Pro (Xiaomi Corporation, Beijing, China), a DJI Osmo Action 5 Pro (SZ DJI Technology Co., Ltd., Shenzhen, China), and a Huawei Pura 70 (Huawei Technologies Co., Ltd., Shenzhen, China). The acquisition device for each author-taken photograph is identified in the corresponding figure caption.

### 2.2. Descriptive Statistical Analysis of the Included Studies

**Figure 3:**
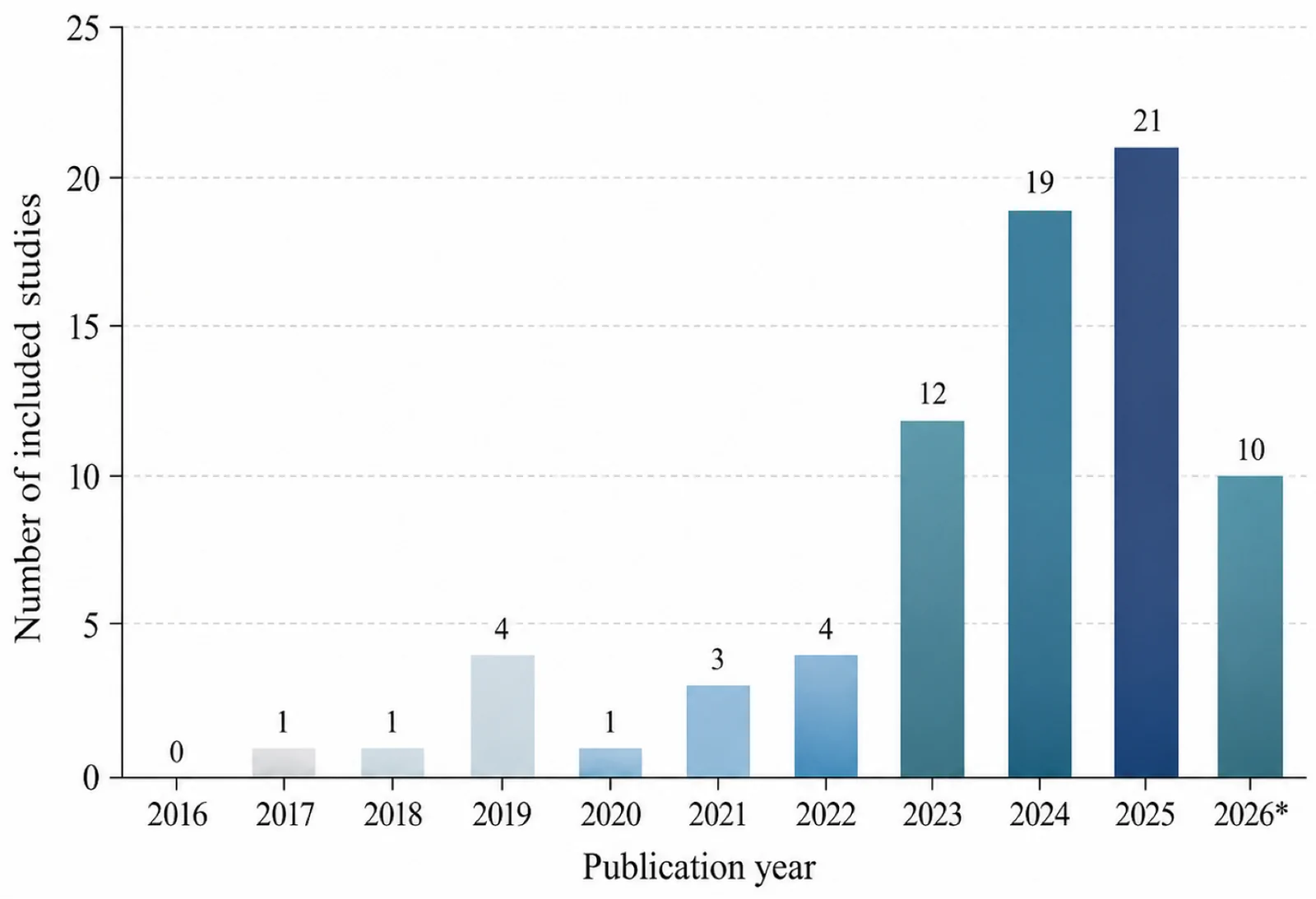
Annual distribution of the core studies included in the descriptive statistical analysis from 2016 to 2026 (n=76). The count for 2026 represents studies available at the time of the final literature search. * indicates that the number of relevant publications for 2026 was counted up to July 2026.

Research on RGB image-based phenotypic analysis of edible mushroom fruiting bodies remained limited during the early part of the study period. No eligible study was identified for 2016, and only a small number were published between 2017 and 2022. The number of publications increased markedly from 2023 onward, reaching 12 studies in 2023, 19 in 2024, and 21 in 2025. The lower count for 2026 should not be interpreted as a decline because the literature search was completed before the end of the year, as shown in Figure 3.

**Figure 4:**
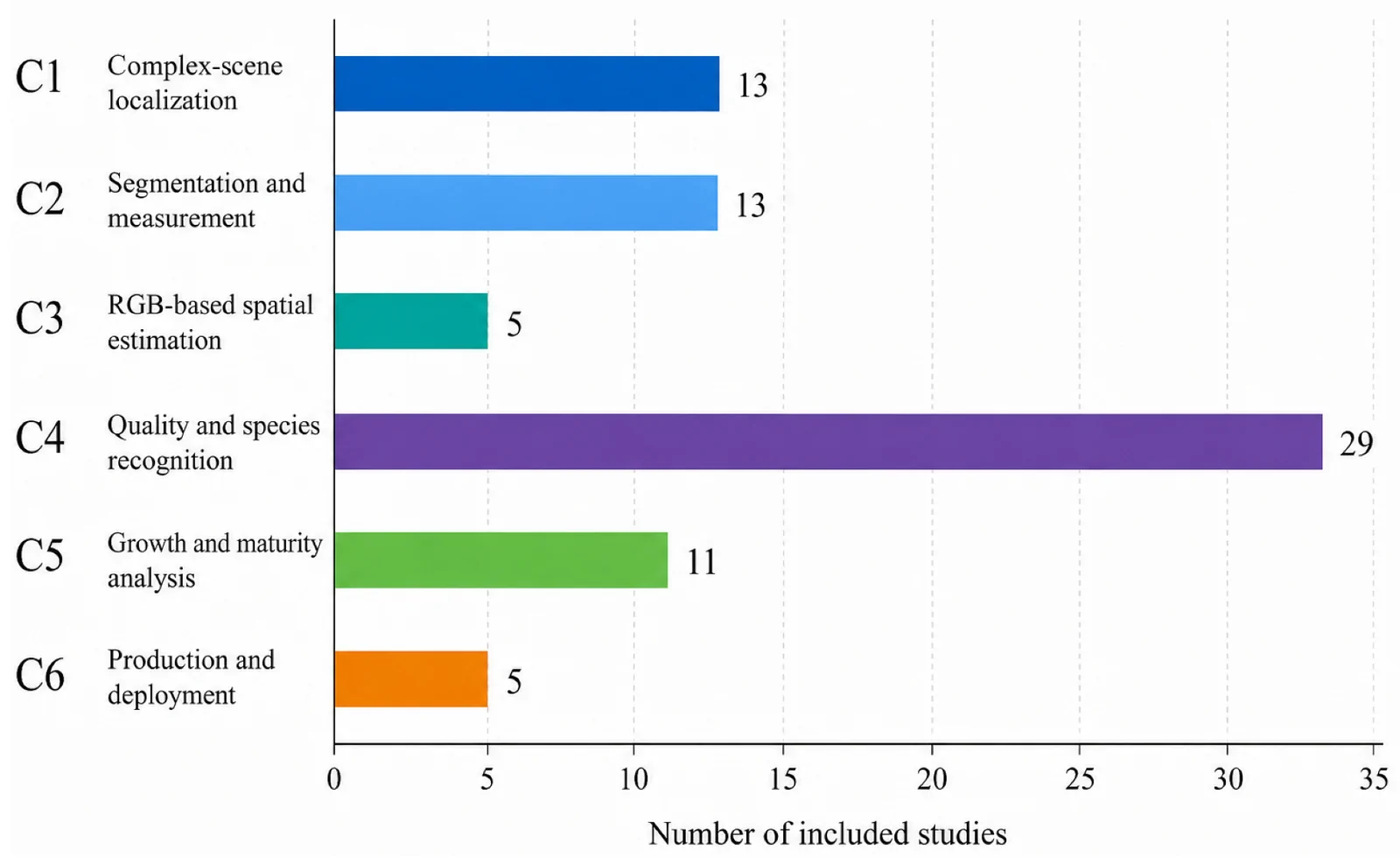
Distribution of the included studies across six major thematic categories. Each study was assigned to one primary category according to its main research objective and methodological contribution (n=76).

The thematic distribution of the 76 core studies was uneven. Appearance-based recognition for quality assessment and species identification constituted the largest category, with 29 studies. Small-object localization and individual separation and structural segmentation, occlusion recovery, and morphological measurement each contained 13 studies, while temporal growth analysis and maturity assessment included 11 studies. Three-dimensional reconstruction and spatial information estimation and production-oriented applications each contained five studies, as summarized in Figure 4.

These distributions indicate that the field has expanded rapidly in recent years but remains unevenly developed. Existing studies are concentrated mainly on appearance recognition, target localization, segmentation, and two-dimensional morphological analysis, whereas RGB-based spatial reconstruction and production-level deployment remain comparatively limited.

## 3. Visible Phenotypes and Visual Tasks

Monocular RGB images mainly capture the visible surface and projected geometric characteristics of edible mushroom fruiting bodies. Based on the information that can be directly or indirectly extracted from such images, relevant phenotypes can be broadly divided into three categories: morphological and structural phenotypes, appearance quality phenotypes, and growth- and maturity-related phenotypes. These categories respectively support quantitative morphology acquisition, commodity quality evaluation, and growth-process assessment. The three phenotype categories form a single dependency chain from image to decision: localization and individual separation determine the target extent, structural and appearance extraction determine what can be measured, and phenotype measurement or state assessment ultimately affects production decisions. Errors at any stage may propagate downstream and become the source of the algorithmic problems examined in Section 4.

### 3.1. Morphological and Structural Phenotype Acquisition

Morphological and structural phenotype acquisition refers to the extraction of quantitative information related to the size, shape, number, location, and spatial distribution of edible mushroom fruiting bodies. At the individual level, commonly used indicators include cap diameter, projected area, perimeter, roundness, cap opening degree, stipe dimensions, and the cap-to-stipe ratio [23,24]. At the population level, representative indicators include visible fruiting-body number, density, coverage, spatial distribution, inter-individual spacing, and occlusion or adhesion relationships [25,26].

These phenotypes are generally derived from bounding boxes, segmentation contours, keypoints, skeletons, and scale-calibrated image measurements [27,28]. Individual size and shape traits can support size grading, morphological selection, and maturity assessment, whereas population-level indicators can provide references for yield estimation, harvesting planning, and production scheduling [29,30]. Object locations and contours can also support mechanical harvesting and robotic localization [31,32].

However, morphological traits derived from monocular RGB images are usually two-dimensional projections or estimates of visible regions rather than complete physical structures. Their reliability is therefore affected by imaging viewpoint, scale calibration, small target size, dense distribution, occlusion, and unclear cap–stipe boundaries [33,34].

Changes in viewpoint directly alter shape and ratio descriptors. On cultivation shelves, differences in tier height and front-to-back position change the camera’s viewing direction relative to each fruiting body, causing different degrees of projective distortion of its three-dimensional structure. For roundness defined by area *A* and perimeter *P* as $C=4\pi A/P^{2}$, an oblique view typically projects a circular cap as an ellipse; area and perimeter no longer vary in the same manner, so the calculated roundness may depart from the value obtained under a frontal view. When roundness is defined by the major-to-minor-axis ratio, both axes are also affected by foreshortening in different directions. The cap-to-stipe ratio is similarly affected: its components lie along different spatial directions, undergo different degrees of foreshortening, and therefore do not cancel through ratio formation. Near a top-down view, stipe shortening or cap occlusion may further shift the ratio. Consequently, the same fruiting body may receive different morphological assessments solely because of viewing angle, whereas pixel-to-millimeter calibration cannot recover pose-induced deformation or invisible structures [8,24,35].

### 3.2. Appearance Quality Phenotypes

Appearance quality phenotypes describe visible characteristics associated with commodity condition, surface integrity, and postharvest quality. Representative features include color and brightness distribution, texture, cracks, spots, decay, contamination, deformity, browning, mechanical damage, and other surface defects [36]. These image characteristics can be converted into color statistics, texture descriptors, defect regions, damage ratios, or quality labels, thereby providing more objective evidence for appearance evaluation than manual visual inspection alone [9,37].

The main applications of appearance quality phenotypes include quality grading, defect detection, postharvest sorting, and abnormality assessment [38,39]. In grading and sorting systems, information related to color, texture, integrity, and local defects can support commodity-grade determination and the removal of non-conforming products [40,41]. Appearance information can also be associated with cultivation or processing conditions, providing a basis for quality tracing and production regulation [42].

Beyond imaging conditions, expansion of the fruiting body itself also alters color and texture statistics. Cap expansion in shiitake mushrooms is accompanied by stage-related changes in surface texture, whereas *Pleurotus pulmonarius* exhibits cap enlargement and color darkening during growth, with adjacent developmental stages potentially being highly similar [43,44]. Consequently, samples of the same quality grade may exhibit different appearance distributions across batches. A model may therefore misinterpret normal development as quality deterioration or rely on batch-specific color-grade associations, and morphological and color changes during stage transitions have been reported to increase recognition errors [45]. Maturity and quality may be related, but normal developmental changes cannot be directly equated with deterioration. Cross-batch quality assessment therefore still requires explicit consideration of growth stage and batch-independent validation.

The capability of monocular RGB images for quality evaluation is nevertheless constrained by illumination variation, imaging angle, surface moisture, background interference, and camera-dependent color differences. Moreover, quality states often vary continuously, while grading standards differ across mushroom species and production scenarios [46]. Consequently, stable quality assessment requires not only visible defect recognition, but also consistent feature extraction and interpretable grading criteria.

### 3.3. Growth- and Maturity-Related Phenotypes

Growth- and maturity-related phenotypes characterize visible changes occurring from primordium formation and fruiting-body expansion to maturity and harvesting. Unlike static morphological traits measured at a single time point, these phenotypes emphasize developmental state, temporal continuity, and the transition toward harvest readiness [47].

A single RGB image can provide maturity-related evidence through cap size, cap opening degree, color, texture, and morphological integrity. Continuous images acquired from fixed viewpoints can further describe changes in fruiting-body number, projected area, population coverage, cap expansion, and mature-individual proportion [48]. These indicators support growth monitoring, maturity assessment, harvest-window prediction, production scheduling, and abnormality warning. Temporal changes in number, area, and coverage can also provide references for evaluating the growth progress of different cultivation batches or regions [49,50].

However, continuous growth assessment depends on stable image acquisition and cross-time association of fruiting bodies. Rapid morphological changes, newly emerging individuals, partial harvesting, dense distributions, occlusion, and ambiguous developmental boundaries may disrupt temporal correspondence and maturity determination [51,52]. The transition from single-frame maturity recognition to temporal growth modeling is therefore examined in detail in Section 4.

### 3.4. From Visible Phenotypes to Production Applications

As summarized in Table 1, the three phenotype categories correspond to measurable morphology, assessable quality, and traceable growth processes. Morphological and structural phenotypes provide the basis for detection, counting, measurement, and harvesting localization. Appearance quality phenotypes support grading, defect recognition, and postharvest sorting, whereas growth- and maturity-related phenotypes support continuous monitoring and harvest decision-making.

The practical value of monocular RGB imaging lies not in acquiring complete biological information, but in providing low-cost, continuously obtainable, and visually interpretable indicators associated with production decisions. Whether these indicators can support yield estimation, grading, maturity assessment, or harvesting depends on their extraction reliability under real cultivation and processing conditions.

This reliability depends on a continuous processing chain: object localization and individual separation → extraction of visible structure and appearance → phenotype measurement or state assessment → production decision. Errors in individual separation affect area, perimeter, roundness, counting, and temporal association. Severe occlusion not only removes contour information but may also hide the stipe and local defects, so failure to detect an abnormality on the visible surface does not prove that the complete fruiting body is normal. When a phenotype depends on invisible structures, the task changes from measurement of visible regions to inference of complete structure, requiring amodal segmentation, structural priors, and geometric constraints; for example, completed masks have been used to estimate the full size of occluded caps [53,54]. Therefore, high detection or segmentation scores do not directly guarantee accurate morphological measurement or stable quality assessment. Section 4 accordingly examines individual separation, structural segmentation and occlusion recovery, three-dimensional spatial estimation, appearance recognition, and temporal modeling, and analyzes the applicability boundaries of these methods under real production conditions.

## 4. Key Bottlenecks and Methodological Evolution

Monocular RGB images provide a low cost data source for phenotypic analysis of edible mushroom fruiting bodies, but the transformation from images to usable phenotypes is not a straightforward process.

Fruiting bodies must first be reliably recognized and localized in complex backgrounds. They then need to be individually separated under dense, occluded, or overlapping conditions, followed by the extraction of structural parameters related to the cap, stipe, and overall morphology. Meanwhile, appearance information such as color, texture, defects, and cap opening degree must also be transformed into quality evaluation and maturity assessment results. Ultimately, these visual phenotypes can demonstrate practical value only when they are linked to real production needs, such as grading, harvesting, yield estimation, and production management.

This section is not organized according to specific model types. Instead, it starts from the key difficulties encountered in fruiting-body phenotypic analysis, summarizes the evolutionary trajectories of existing methods, and analyzes their limitations in real production applications.

### 4.1. Small-Object Localization and Individual Separation

**Figure 5:**
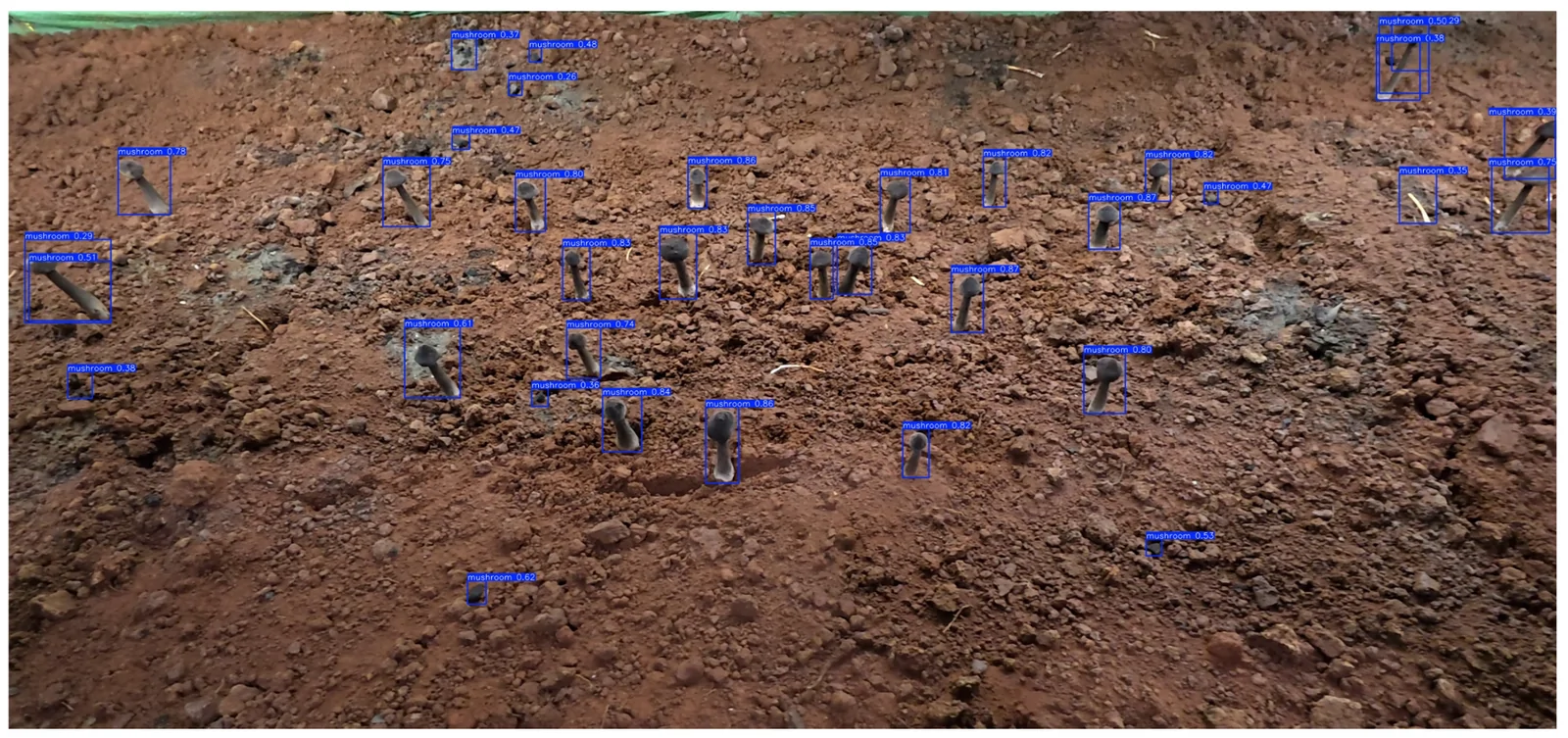
Early-stage *Oudemansiella raphanipes* fruiting bodies with small scales, uneven distribution, local crowding, and substrate interference. Photograph acquired by the authors using a Canon EOS 90D.

Object localization and individual separation form the basis of image-based phenotypic analysis because errors at this stage propagate to counting, morphological measurement, maturity assessment, quality evaluation, and harvesting localization. In cultivation scenes, early-stage fruiting bodies may occupy only a few pixels, vary greatly in scale, and appear either sparsely or in locally crowded groups against irregular substrates. These conditions increase missed detections, false detections, duplicate detections, and localization errors. The early-stage *Oudemansiella raphanipes* scene in Figure 5 exemplifies these challenges through weak target–background contrast, uneven distribution, and substantial scale variation.

Existing detection methods mainly improve shallow-detail preservation, multi-scale feature fusion, contextual representation, and bounding-box regression. MSH-YOLOv8 uses feature-scale reconstruction and multi-scale fusion for small mushroom targets [55], while Recursive YOLOv5 combines recursive structures, atrous spatial pyramid pooling, and DIoU-NMS for high-resolution cultivation-stick scenes [56]. An improved RT-DETR further integrates FasterNet, feature-fusion modules, SPDConv, and EIoU to balance small-target accuracy and efficiency under low illumination [57]. For dense populations, improved YOLOv7 with AFPN and MDIoU reduces localization errors [58]; YOLOv5s-CBAM with Mosaic augmentation supports detection, counting, center localization, and approximate size measurement of *Agaricus bisporus* [59]; and an RFBSE–DCSPP–RFP-enhanced YOLOv5s strengthens edge and contextual features in complex *Oudemansiella raphanipes* scenes [60]. These approaches improve target visibility and localization, but bounding boxes still provide only approximate extents and cannot explicitly separate individuals whose visible regions overlap or adhere.

Instance segmentation therefore represents the main transition from approximate localization to pixel-level individual separation. YOLOv5seg-BotNet combines BoTNet, PANet, and Varifocal Loss for shiitake instance segmentation [61], whereas PR-SOLOv2 introduces PointRend and contour reconstruction to refine boundaries and centers in dense clusters [62]. Comparative evaluations of Mask R-CNN, Cascade Mask R-CNN, Hybrid Task Cascade, and DetectoRS have examined framework suitability across natural and farm environments [63], and related pipelines have extended instance masks to continuous acquisition and cross-time tracking [64]. For robotic harvesting, SCT-Mask-RCNN integrates spatial-channel attention and multi-scale fusion to provide contours and localization cues for manipulation [65]. Evaluation reliability, however, also depends strongly on dataset partitioning and mask annotation quality [66].

Representative studies on small-object localization and individual separation, together with their methodological characteristics, reported performance, and major limitations, are summarized in Table 2.

Methodologically, this field has progressed from small-target-sensitive detection toward pixel-level instance separation. Detection remains efficient for approximate localization and counting, whereas instance segmentation is more suitable for dense populations and downstream phenotype measurement. Both routes, however, remain sensitive to weak texture, irregular substrates, illumination variation, scale differences, invisible boundaries, and annotation quality. Reliable separation at this stage is therefore a prerequisite for subsequent structural segmentation and morphological measurement, while recovery of anatomically complete but invisible structures requires additional occlusion-aware modeling.

### 4.2. Structural Segmentation, Occlusion Recovery, and Morphological Measurement

Structural segmentation and fine-grained morphological measurement represent a critical step in advancing image-based phenotypic analysis of edible mushrooms from object recognition to quantitative trait extraction. After detection and individual separation, the cap, stipe, and whole fruiting body must be delineated before quantitative traits can be derived. Cap-related indicators include diameter, projected area, perimeter, roundness, and cap height [8,24], whereas stipe-related indicators include length, thickness, centerline, and curvature [67]. Whole-fruiting-body dimensions and their temporal changes can further support growth measurement and phenotypic monitoring [25]. These traits are relevant to breeding selection, size grading, quality evaluation, growth assessment, and harvesting decisions.

**Figure 6:**
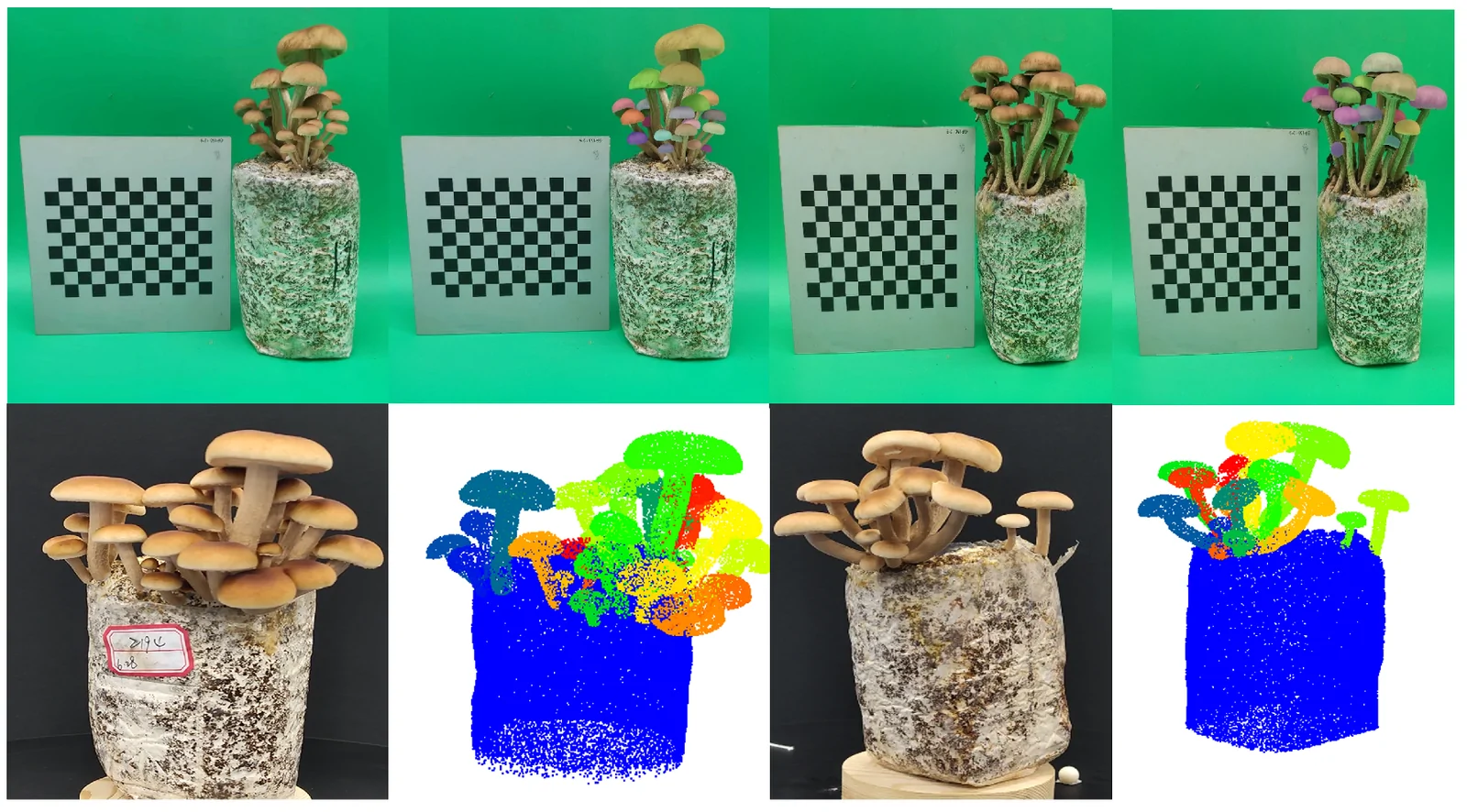
Two-dimensional segmentation and three-dimensional point-cloud representation of *Oudemansiella raphanipes* under dense cultivation conditions. The upper row shows 2D segmentation results, whereas the lower row presents corresponding RGB images and 3D point-cloud representations, illustrating mutual overlap and partial occlusion among adjacent fruiting bodies.

Occlusion is particularly important at this stage because the visible region of a fruiting body may represent only part of its complete anatomical structure. In densely cultivated populations, caps frequently overlap one another, while stipes may be partially hidden by caps or adjacent individuals. As illustrated in Figure 6, dense growth, contact between adjacent fruiting bodies, and front–back overlap can lead to incomplete cap and stipe contours. Direct measurement from such visible regions may underestimate cap diameter, area, perimeter, stipe length, and complete fruiting-body height. Therefore, the problem is no longer limited to determining whether a fruiting body is present, but extends to recovering structurally meaningful boundaries from incomplete visual evidence.

**Figure 7:**
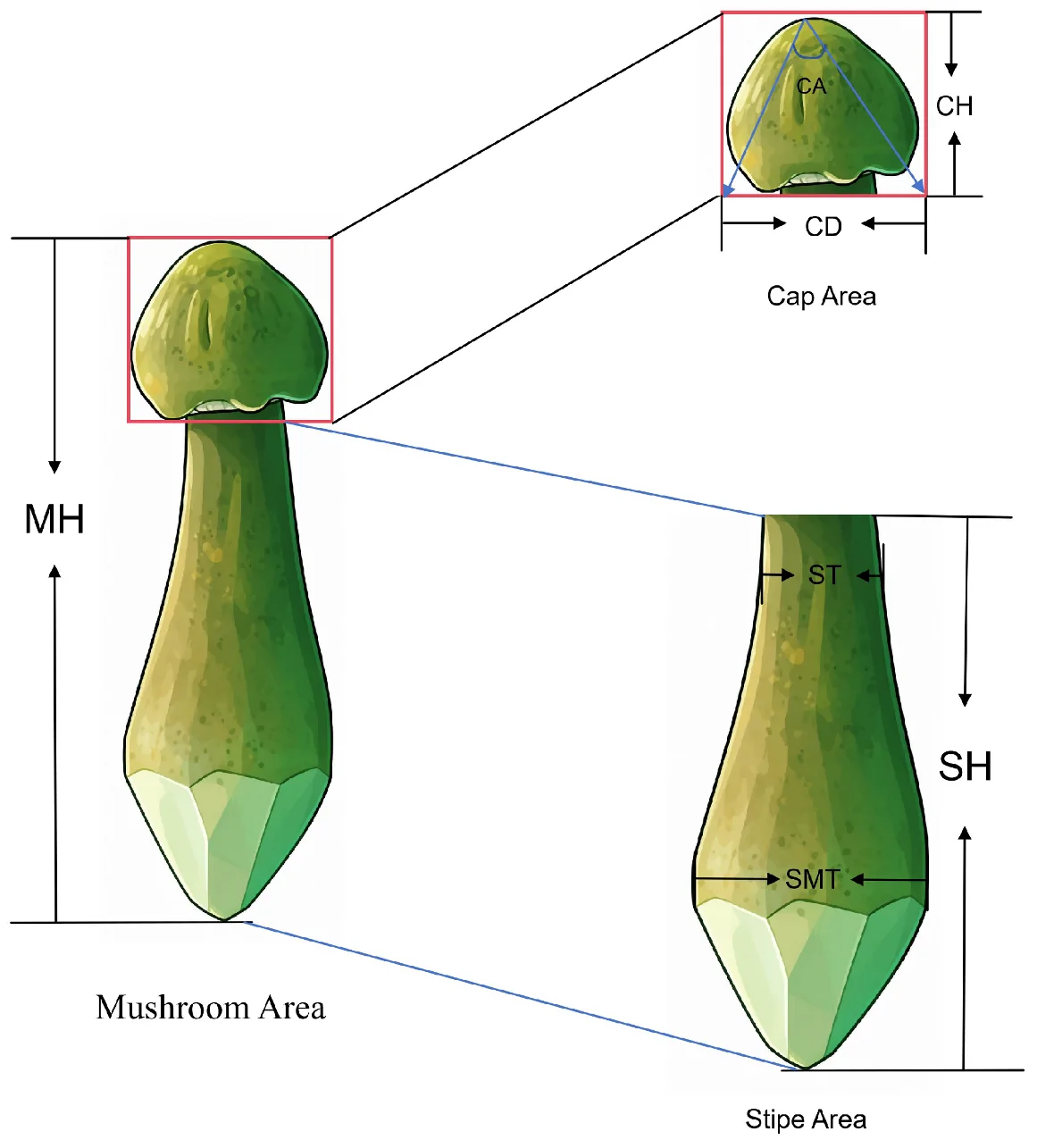
Schematic illustration of structural segmentation and representative morphological traits of an edible mushroom fruiting body. MH, mushroom height; CH, cap height; CD, cap diameter; SH, stipe height. Underlying photograph acquired by the authors using a Redmi K20 Pro.

Under relatively complete visibility, structural segmentation provides the basis for distinguishing the cap, stipe, and whole fruiting-body regions. As shown in Figure 7, different morphological traits depend on different anatomical regions and boundary definitions. The whole fruiting-body region can be used to estimate mushroom height and projected area, whereas the cap region provides traits such as cap diameter, cap height, and cap area. The stipe region supports the estimation of stipe height, area, thickness, and centerline. Reliable measurement therefore depends not only on accurate fruiting-body localization, but also on stable extraction of the cap contour, stipe contour, and cap–stipe junction.

Early studies mainly combined object localization, traditional image processing, and geometric rules to measure visible morphological structures. Lu et al. [25] developed a growth measurement system in which CNN-based localization and position correction were used to record mushroom number and size for growth-rate estimation. Subsequently, CNN-based recognition was combined with a Score-Punishment algorithm to estimate the diameter of approximately circular caps, reducing the strong dependence of conventional Hough circle detection on manually selected parameters [23]. For fresh *Agaricus bisporus*, Wang et al. [68] used threshold segmentation, watershed processing, Canny edge detection, and morphological operations to suppress shadows and stipe interference before extracting geometric features such as maximum diameter. In high-throughput breeding analysis, grayscale conversion, binarization, contour extraction, and cap attachment detection were used to quantify morphological traits of detached and scanned *Flammulina velutipes* samples [69]. These methods are computationally simple, but their performance generally depends on clear foreground–background contrast, complete contours, and relatively controlled imaging conditions.

With the development of deep segmentation and edge-detection models, research has shifted from rule-based contour extraction toward automated structural region delineation. Choi et al. [70] compared YOLOv8-seg and YOLOv11-seg for cap and stipe segmentation and validated image-derived measurements against manual caliper measurements, demonstrating the feasibility of segmentation-based digital phenotyping under controlled cultivation conditions. Zhou and Wang [71] applied an improved Mask R-CNN to segment the cap, stipe, and crack regions of *Pleurotus geesteranus*, enabling both structural trait extraction and visible damage quantification. For shiitake caps, Zhao et al. [24] developed a KL-DexiNed edge-detection model and mapped the detected edges back to the original image before using OpenCV 4.14.0 to calculate cap area, perimeter, and bounding-rectangle dimensions.

Fine-grained stipe measurement presents additional difficulties because stipes are slender, frequently curved, and easily obscured by the cap or neighboring fruiting bodies. Bounding-box dimensions cannot reliably represent stipe length, thickness variation, centerline, or curvature. To address this problem, Zhao et al. [67] combined an ACmix-ADown-YOLOv11n detector with EfficientSAM-based segmentation and OpenCV-based calculation to extract multiple stipe traits. A pose-estimation model was further introduced to predict the stipe centerline, extending structural measurement from region-level dimensions toward curved anatomical geometry.

However, visible-region segmentation alone remains insufficient when substantial parts of the cap or stipe are occluded. This limitation has motivated the use of amodal segmentation, synthetic occlusion data, structural priors, and camera geometry. Wei et al. [54] generated synthetic occlusion images, constructed an amodal instance segmentation dataset, and estimated complete cap length and width from predicted amodal masks. This approach enabled phenotype extraction even when only part of the cap was directly visible. For occluded *Oudemansiella raphanipes* caps, Yin et al. [53] proposed OR-ORCNN with contextual attention and multi-scale feature fusion, and combined the predicted amodal mask with a pinhole camera model to estimate complete cap dimensions. Tao et al. [8] incorporated cultivation-stick geometry and monocular visual detection to estimate shiitake cap size, roundness, and the proportion of qualified mushrooms for selective harvesting evaluation. These methods extend conventional segmentation from visible contour extraction to complete-shape inference, although their accuracy depends on the validity of occlusion simulation, anatomical assumptions, camera calibration, and the amount of invisible structure.

Table 3 summarizes representative studies on structural segmentation, occlusion recovery, and morphological measurement.

Existing methods demonstrate a clear progression from measurement based on visible contours toward semantic or instance-level structural segmentation and complete-shape inference under occlusion. Nevertheless, three limitations remain evident. First, measurements derived from two-dimensional projected regions do not necessarily correspond to true physical dimensions, particularly when fruiting bodies are inclined, curved, or viewed obliquely. Second, cap diameter, roundness, stipe length, thickness, and curvature are not defined or calculated consistently across studies, reducing the comparability of reported phenotypic results. Third, amodal recovery can estimate invisible structures, but the recovered morphology remains model-dependent rather than directly observed. Consequently, the reliability of phenotype extraction depends jointly on segmentation accuracy, boundary visibility, structural assumptions, imaging geometry, and the specific trait being measured [70,72].

### 4.3. RGB-Based Three-Dimensional Reconstruction and Spatial Estimation

Most phenotypic traits extracted from monocular RGB images are based on two-dimensional projections, which cannot directly represent fruiting-body volume, absolute depth, stipe orientation, surface curvature, or complete spatial relationships under occlusion. These traits are relevant to yield estimation, growth analysis, and robotic harvesting localization. Although RGB-D cameras and dedicated depth sensors can directly acquire depth maps or point clouds, their cost, calibration requirements, and deployment complexity may restrict large-scale application. Recovering or approximating spatial information from ordinary RGB images therefore provides a low-cost extension of conventional two-dimensional phenotyping.

Existing studies mainly follow three routes: multi-view RGB reconstruction, monocular depth prediction, and geometry-assisted spatial inference. These routes differ in acquisition requirements, output form, metric reliability, and dependence on prior assumptions, as summarized in Table 4.

Multi-view RGB reconstruction recovers three-dimensional structures from correspondences among images acquired at different viewpoints. As a boundary extension of the monocular RGB scope of this review, Xu et al. [73] acquired smartphone images of shiitake fruiting bodies from multiple heights and viewing angles and combined Structure-from-Motion and Multi-View Stereo for point-cloud reconstruction, three-dimensional trait extraction, and yield estimation. This route can provide relatively complete and metrically meaningful spatial representations, but requires sufficient viewpoint coverage, surface texture, image correspondence, and point-cloud post-processing.

Monocular depth prediction estimates spatial information directly from a single RGB image, and is therefore more compatible with low-cost fixed-camera systems. Wang et al. [74] developed a multi-task model for oyster mushroom instance segmentation and depth prediction, obtaining fruiting-body regions and relative depth cues without a dedicated depth sensor. Predicted depth changes were further used to characterize growth. However, monocular depth generally contains scale ambiguity and may be sensitive to species, illumination, viewpoint, background, and cultivation conditions.

Geometry-assisted methods introduce camera models or structural priors into two-dimensional analysis rather than reconstructing complete three-dimensional surfaces. Amodal segmentation and pinhole camera geometry have been used to estimate the complete dimensions of partially occluded *Oudemansiella raphanipes* caps [53]. Cultivation-log relationships and monocular geometric inference have supported shiitake cap-size estimation, roundness evaluation, and selective harvesting assessment [8]. Synthetic occlusion and amodal instance segmentation have also enabled complete cap-shape recovery from incomplete visible regions [54]. These methods can reduce errors caused by occlusion, but their outputs depend on camera geometry, orientation assumptions, synthetic occlusion patterns, and learned shape priors. Moreover, recovered masks remain two-dimensional shape estimates and do not directly provide thickness, curvature, volume, or absolute depth.

The three routes should not be regarded as equivalent forms of three-dimensional phenotyping. Multi-view reconstruction can recover point clouds and metric spatial traits but requires more demanding acquisition and processing. Monocular depth prediction is easier to deploy but generally provides relative rather than metrically accurate depth. Geometry-assisted inference improves two-dimensional measurement under occlusion but does not reconstruct complete three-dimensional morphology. RGB-based spatial phenotyping of edible mushrooms therefore remains exploratory, particularly in terms of metric accuracy, cross-scene generalization, and validation against calibrated depth or point-cloud references.

### 4.4. Appearance-Based Quality and Species Recognition

Appearance-based recognition from monocular RGB images mainly supports appearance quality assessment and species identification. Both tasks rely on visible color, texture, shape, surface integrity, and cap–stipe structure, but their objectives differ. Quality assessment evaluates commercial condition within a species or product category, whereas species identification determines mushroom identity, and may further support edible–poisonous discrimination. The former is closely related to grading, sorting, and quality control, while the latter serves category recognition, food safety, and resource investigation.

**Figure 8:**
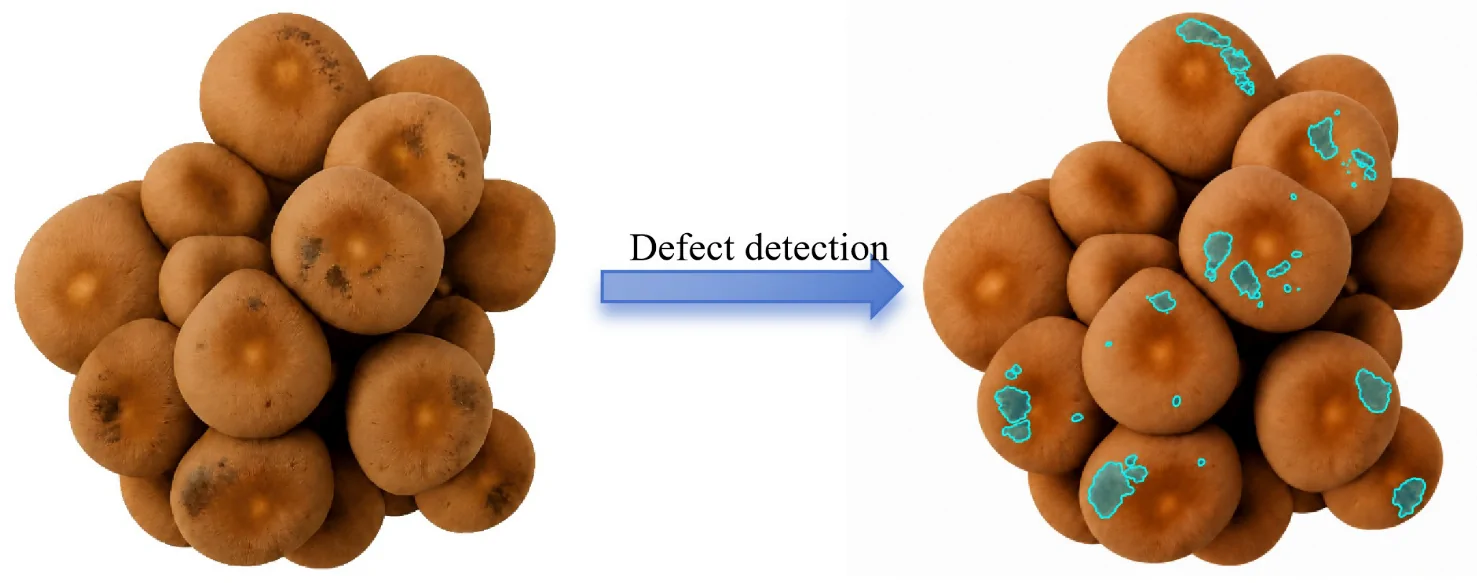
Mushroom fruiting bodies exhibiting surface damage and visible defects. Photographs acquired by the authors using a Redmi K20 Pro.

Appearance quality assessment converts visible attributes, including color, texture, integrity, deformity, cracks, damage, browning, maturity, and freshness, into grades, defect labels, or processing indicators. As illustrated in Figure 8, these attributes may occur simultaneously and can be affected by occlusion and local appearance variation.

Existing methods can be grouped into three routes: discrete grade classification, defect localization, and continuous quality estimation. Early classification studies used relatively simple convolutional networks, such as an improved LeNet for *Flammulina velutipes* cap grading [75]. Subsequent methods combined segmentation, deeper feature extraction, and lightweight detection. OOA-optimized Otsu segmentation and D-VGG were used to distinguish six grades of dried shiitake mushrooms [76]; improved YOLOX with channel pruning and knowledge distillation supported efficient shiitake quality recognition [37]; and OMC-YOLO introduced lightweight convolution, attention, neck optimization, and localization loss for oyster mushroom grading [41]. More production-oriented studies connected visual grading with air-jet sorting through YOLOv8-seg, OpenCV, and SegGrade [38], or jointly modeled detection, maturity, and grade classification using Mamba-YOLO [9].

Defect-oriented methods focus on subtle and localized changes rather than only assigning an overall grade. OMB-YOLO and its pruned OMB-YOLO-tiny variant balanced oyster mushroom damage detection and deployment efficiency [77], while CMCI-RTDETR enhanced the localization of cracks, boundaries, and small defects on shiitake caps through multi-scale edge and contextual features [78]. Other studies quantified continuous quality changes: online image analysis measured wrinkling and radial shrinkage during shiitake drying [79], neural-network-based estimation characterized freshness deterioration using the Hedonic number [80], and detection plus segmentation was used to calculate the browning-area ratio of shiitake cultivation sticks [81].

These studies show a progression from discrete classification toward defect localization, quantitative quality indicators, lightweight deployment, and physical sorting. However, quality changes are often continuous, whereas most models use a small number of discrete labels. Definitions of quality, damage, maturity, browning, and freshness also vary among studies, and subtle defects remain sensitive to illumination, imaging distance, surface moisture, camera settings, and segmentation errors. Cross-variety, cross-batch, cross-device, and long-term production-line validation therefore remains limited.

The absence of harmonized metrics for browning, spots, and micro-cracks is an increasingly urgent obstacle to commercial comparability. Browning may be represented as a classification label, CIELAB color change, or browning-area ratio [37,81]; spot severity may be reported as count, number density, affected-area fraction, or a subjective grade; and micro-crack severity may vary with crack length density, width, connectivity, and image resolution [77,78]. Because these endpoints are not equivalent, identical accuracy values may be based on incompatible definitions. A unified reporting protocol should distinguish spatial extent, intensity, and morphology, and should state the reference standard, threshold source, segmentation denominator, growth stage, surface-moisture condition, color calibration procedure, and inter-rater agreement. Thresholds should be traceable to physical measurements, official standards, or an expert-defined protocol rather than remaining study-specific. Until such harmonization is achieved, reported grading accuracy should be interpreted as dataset- and threshold-specific rather than directly commercially comparable.

**Figure 9:**
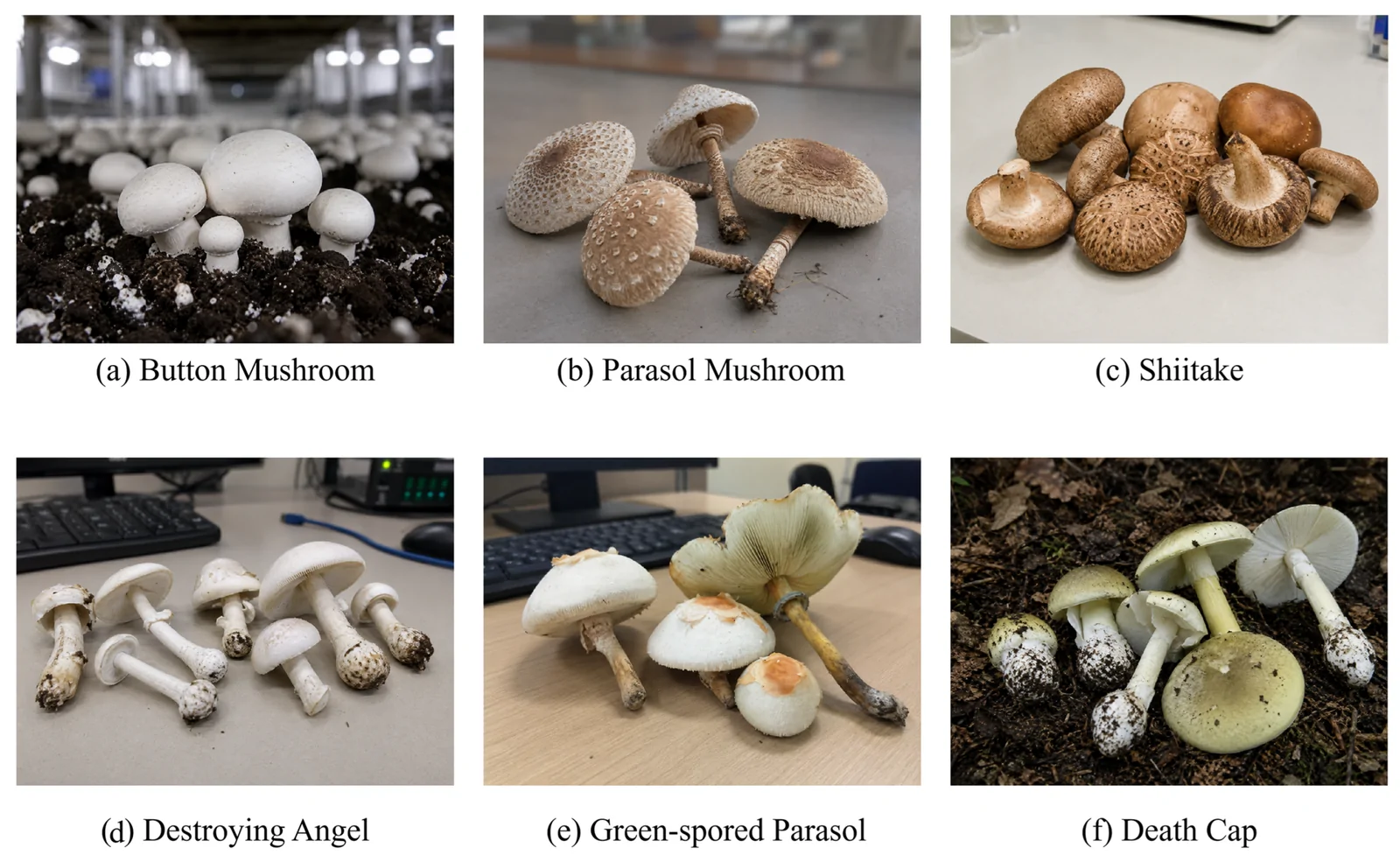
Examples of mushroom species identification based on visible appearance characteristics. Photographs acquired by the authors using a Huawei Pura 70.

In addition to quality assessment, appearance-based recognition is also widely used for species identification and safety-oriented classification. Unlike quality grading, which evaluates appearance variation within a species or commercial category, species identification aims to distinguish among mushroom categories that may exhibit substantial visual similarity. This is a fine-grained recognition problem because different species may share similar cap colors, textures, gill patterns, and stipe structures, while the same species may vary across growth stages, regions, habitats, viewpoints, and illumination conditions. Representative edible and poisonous mushrooms exhibiting diverse visible characteristics are shown in Figure 9.

Earlier edible–poisonous classification studies mainly relied on structured morphological or descriptive attributes rather than direct RGB images. Conventional classifiers, including K-nearest neighbors, decision trees, Random Forest, Logistic Regression, and Naive Bayes, were comparatively evaluated by Chitayae and Sunyoto [82]. Feature selection and hyperparameter optimization were subsequently introduced to improve attribute-based classification [83], while the discriminative contribution of selected descriptive variables was further examined by Ortiz-Letechipia et al. [84]. Although these approaches provide interpretable baselines, they cannot fully represent image-level variations in background, pose, occlusion, and acquisition quality, and are therefore not equivalent to direct RGB-image recognition.

Image-based studies subsequently adopted convolutional networks and transfer learning. InceptionV3, VGG16, and ResNet50 with contrast enhancement were compared for edible, inedible, and poisonous classification [85]; improved AlexNet was evaluated for Thai edible and poisonous mushrooms [86]; and residual transfer learning was used to strengthen texture and local-detail representation [87]. Research then expanded toward multi-species and regional recognition using DenseNet, MobileNetV2, and InceptionResNetV2 [88], locally collected Bangladeshi datasets [89], and Malaysian datasets evaluated with Vision Transformer and ResNet models [90]. These studies improved coverage of regional species but also exposed limited transferability across habitats and geographic distributions.

Recent methods have emphasized fine-grained and multi-scale representation. ResNet combined with region-based CNNs was used to analyze inter-class confusion in wild mushroom recognition [91]; EfficientNetB7 revealed substantial class-wise differences in precision and recall [92]; and AWPF-ResNet18 combined adaptive window pyramid fusion with dynamic Swin windows to enhance cap texture and stipe morphology features [93]. Nevertheless, single-view RGB images may omit diagnostic structures such as gills, volvas, rings, or stipe bases. Models may also exploit dataset-specific backgrounds rather than biological traits, and most studies remain limited to closed-set categories. Cross-regional testing, unknown-category rejection, confidence calibration, and risk-sensitive evaluation are therefore essential before image classification can support food-safety decisions. Representative methods for both appearance quality assessment and species identification are summarized in Table 5.

Quality assessment and species identification share the need for stable appearance features under changes in illumination, pose, background, and imaging devices, but their evaluation priorities differ. Quality assessment requires continuous or ordinal representation, cross-batch consistency, and production-line validation, whereas species identification requires cross-regional generalization, unknown-category recognition, and explicit control of safety-critical errors. For quality assessment, harmonized quantitative definitions of browning, spots, and micro-cracks are also required so that results can be compared directly across studies, devices, and production lines. These distinctions should guide the translation of RGB recognition models into production and food-safety applications.

### 4.5. Temporal Growth and Maturity Assessment

**Figure 10:**
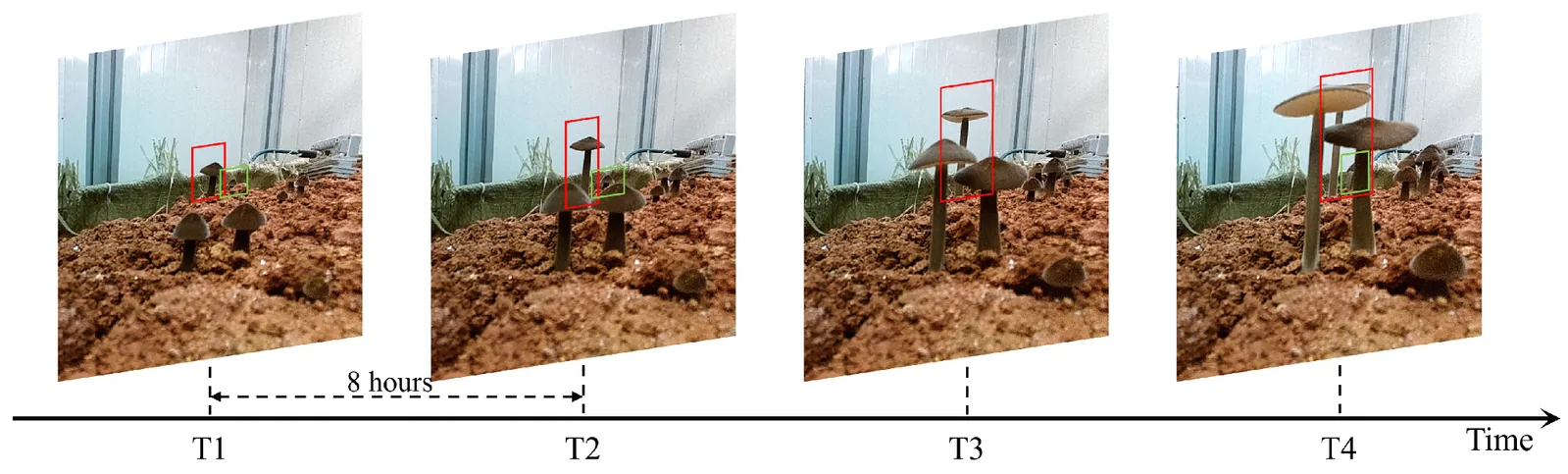
Representative time-lapse sequence of edible mushroom fruiting-body development. The red and green boxes highlight example fruiting bodies whose visibility changes owing to progressive occlusion. Image sequence acquired by the authors using a DJI Osmo Action 5 Pro.

Maturity and growth-stage assessment is an important task in image-based phenotypic analysis of edible mushrooms for continuous growth monitoring and harvest decision-making. Figure 10 presents a representative time-lapse sequence of the same cultivation scene during fruiting-body development. Across successive observations, the fruiting bodies exhibit clear stem elongation and cap expansion, while the spatial relationships among neighboring individuals continuously change.

The red and green boxes highlight two example fruiting bodies that become progressively occluded during development. The instance marked by the green box becomes completely invisible in the third frame and only a small visible part reappears in the fourth frame, whereas the instance marked by the red box is also almost fully occluded by the enlarged cap of a neighboring fruiting body in the fourth frame. This example illustrates that temporal analysis involves not only describing growth dynamics, but also maintaining cross-frame correspondence of the same individuals under dense clustering, scale change, and increasing occlusion.

One common methodological route is to formulate maturity and growth-stage assessment as a visual detection or segmentation problem, in which fruiting bodies at different developmental stages are identified to support harvesting and environmental regulation. For example, Moysiadis et al. [94] used YOLOv5 to detect growth stages of pleurotus mushrooms and combined Detectron2 for growth monitoring, estimating the average growth period and assisting harvest-date determination, thereby providing a reference for vision-based mushroom growth-status monitoring. Wang et al. [44] proposed a lightweight GSP-RTMDet model for mushroom-house scenarios of *Pleurotus pulmonarius*, incorporating SPPF-SAM, CSP-SGE, and CARAFE modules to achieve real-time growth-stage detection and instance segmentation, providing visual evidence for growth-stage-based mushroom-house environmental control. Xu et al. [95] addressed the subtle differences in shiitake maturity states by proposing YOLOv8n-CFS, which integrates SegNeXt Attention, a feature diffusion propagation network, global cue reasoning, and fine-grained spatial information modeling to improve shiitake maturity detection in complex environments.

Beyond single-frame maturity assessment, time-series images provide a more direct information source for continuous growth monitoring of edible mushrooms. Charisis et al. constructed an instance segmentation and tracking pipeline based on Mask R-CNN, using ConvNeXt or Swin backbones to detect and segment oyster mushroom clusters, and further implemented cross-time image tracking and area estimation based on instance masks to characterize the continuous growth process of mushroom clusters [26]. Nuwayhid et al. focused on multi-view time-series images of oyster mushroom clusters, using Mask R-CNN with a Swin backbone for instance segmentation and characterizing growth changes from different viewpoints through temporal tracking and relative area curves [64]. These studies indicate that maturity assessment can be extended from single-frame classification to continuous tracking. However, under early-stage small targets, weak illumination, occlusion, and partial harvesting, instance consistency and area estimation remain vulnerable.

On the basis of continuous monitoring, some studies have further attempted to predict future maturity time or growth status. Fan et al. addressed the limitation that many existing vision-based methods remain at the level of growth-stage classification and cannot predict the time to full maturity. They proposed the YOLO-P detection-prediction framework, which integrates Swin Transformer v2 into YOLOv12 to enhance long-range feature modeling and combines logistic curve fitting with recursive Gaussian fusion to predict the full maturity time of gray oyster mushrooms [45]. Xu et al. proposed the VMRNN-DMSA spatiotemporal prediction model, incorporating skip connections, Max Feature Map, spatial attention, and adaptive convolution modules into the Vision Mamba RNN Depth architecture to improve the accuracy and image quality of long-term growth prediction for shiitake fruiting bodies [43]. These studies advance the task from current-stage recognition to future-state prediction, which is more closely aligned with practical requirements for production scheduling and harvest planning.

Most temporal models implicitly treat elapsed time as a proxy for biological development. This assumption is fragile in protected cultivation because temperature and relative-humidity fluctuations alter water status, metabolic activity, and the trajectory of cap expansion [49,96]. Consequently, the same calendar interval may correspond to different visible developmental states across batches, and a trajectory learned under one environmental regime may systematically lead or lag under another. Fixed-interval labels can therefore conflate time with biological state. Current RGB studies generally record timestamps and growth-stage labels but rarely incorporate continuous temperature, relative humidity, or vapor-pressure deficit as time-varying inputs [12,42]. Environment-aware models should use lagged or cumulative environmental variables and should report whether this feedback improves cross-batch maturity prediction. Until such validation is available, time-only predictions should be interpreted as condition-specific trajectories rather than environment-independent biological clocks.

This line of research has progressively evolved from single-frame detection and classification toward instance segmentation, temporal tracking, maturity-time prediction, primordium number estimation, and real-time interpretable monitoring. Different methods emphasize different research objects, task objectives, and technical routes. Some focus on current maturity status recognition, some focus on continuous growth trajectories, and others further predict full maturity time or support intelligent sorting. To facilitate comparisons of different studies in terms of task type, core method, and major contributions and limitations, Table 6 summarizes representative studies in this area.

Monocular RGB imaging has been increasingly applied to maturity recognition, growth-stage detection, and continuous growth monitoring of edible mushrooms. Existing methods have gradually progressed from single-frame stage classification and object detection toward instance segmentation, temporal tracking, and maturity prediction. Representative studies and their methodological characteristics are summarized in Table 6.

Despite this progress, production-oriented growth understanding remains constrained by several common limitations. Many studies still rely primarily on single-frame classification or detection, which cannot fully represent the continuous and nonlinear development of fruiting bodies. Maturity labels are often defined according to manual experience or scenario-specific production standards, and their meanings may vary across mushroom species, cultivation modes, and harvest objectives, limiting the comparability of model outputs. Temporal tracking and maturity prediction are more closely aligned with production scheduling, but their performance remains sensitive to occlusion, illumination variation, partial harvesting, newly emerging fruiting bodies, and the disappearance of existing individuals. Consequently, cross-time instance consistency, standardized maturity representation, and long-term prediction stability remain insufficiently addressed. In addition, fixed-interval models implicitly assume that elapsed time and developmental progress remain synchronized, whereas temperature and relative-humidity fluctuations may alter cap expansion, surface changes, and the progression toward maturity. To reduce cross-batch drift, models should incorporate continuous environmental records as time-varying covariates, test whether environmental feedback improves prediction, and report performance under both controlled and uncontrolled environmental conditions. The transition from single-frame appearance recognition to continuous, stable, and interpretable growth-state modeling therefore remains a central challenge in RGB-based mushroom phenotyping.

### 4.6. Production Applications and Deployment Challenges

Transferring image-based edible mushroom phenotyping from laboratory studies to production environments remains a system-level challenge. Although detection, segmentation, morphological measurement, quality grading, species recognition, and maturity assessment have achieved promising results, most evaluations rely on specific datasets or relatively controlled acquisition conditions. Real production environments introduce illumination variation, complex backgrounds, occlusion, batch and device differences, viewpoint changes, and workflow constraints.

Recent studies have extended RGB-based phenotyping to high-throughput breeding analysis [69], fixed-camera monitoring [98], mobile recognition [99], conveyor-belt grading [100], and robotic harvesting [101]. Representative production-oriented studies, together with their system configurations and practical limitations, are summarized in Table 7.

**Figure 11:**
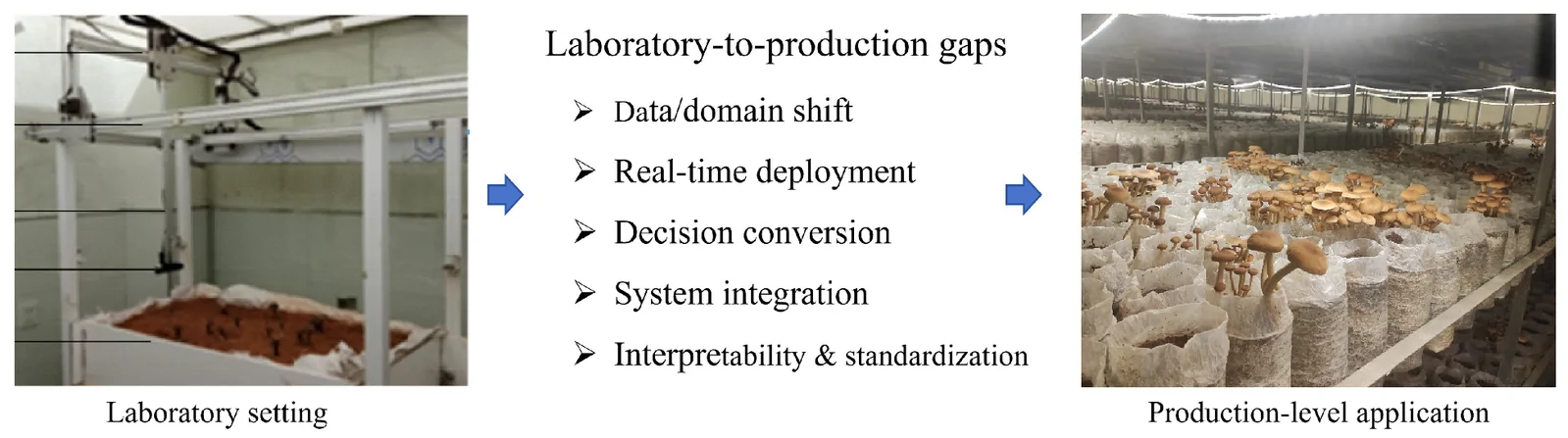
Major bottlenecks in translating image-based edible mushroom phenotyping from laboratory settings to production-level applications. Photographs acquired by the authors using a Huawei Pura 70.

Despite these advances, the transition from laboratory recognition to sustained production use remains constrained by data and domain shift, real-time deployment, decision conversion, system integration, and insufficient interpretability and standardization. In addition, the economic feasibility for small and medium-scale producers and the match between edge-device performance and production cycle time remain two deployment conditions for which quantitative evidence is generally lacking. These gaps are illustrated in Figure 11.

Minimum perception rate should be derived from process kinematics, $f_{\min}\geq nq$, where *q* is the target arrival rate and *n* is the number of independent observations per target. For example, 1066.67 mushrooms/min corresponds to q=17.78 mushrooms/s; the theoretical minimum is 17.78 FPS when n=1 and 53.33 FPS when n=3. Production studies should report device model, input resolution, precision mode, batch size, absolute FPS, p50/p95 end-to-end latency, frame loss, throughput, actuator response, RH range, protection rating, and cleaning or defogging intervals. High humidity primarily affects condensation, image degradation, contamination, and maintenance downtime rather than directly defining a universal FPS threshold.

The first deployment gap is cross-scenario generalization. Mushroom images vary with species, cultivation mode, substrate, growth stage, illumination, imaging device, and production site, so models trained on one dataset may degrade across batches, mushroom houses, or acquisition platforms. This issue occurs across standardized breeding images and mushroom-house CCTV data [69,98], complex ecological scenes for toxic mushroom detection [102], and mobile-oriented species-recognition systems [99]. Production deployment therefore requires evaluation across independent sites, batches, devices, and operating conditions rather than only random image-level train–test splits.

**Figure 12:**
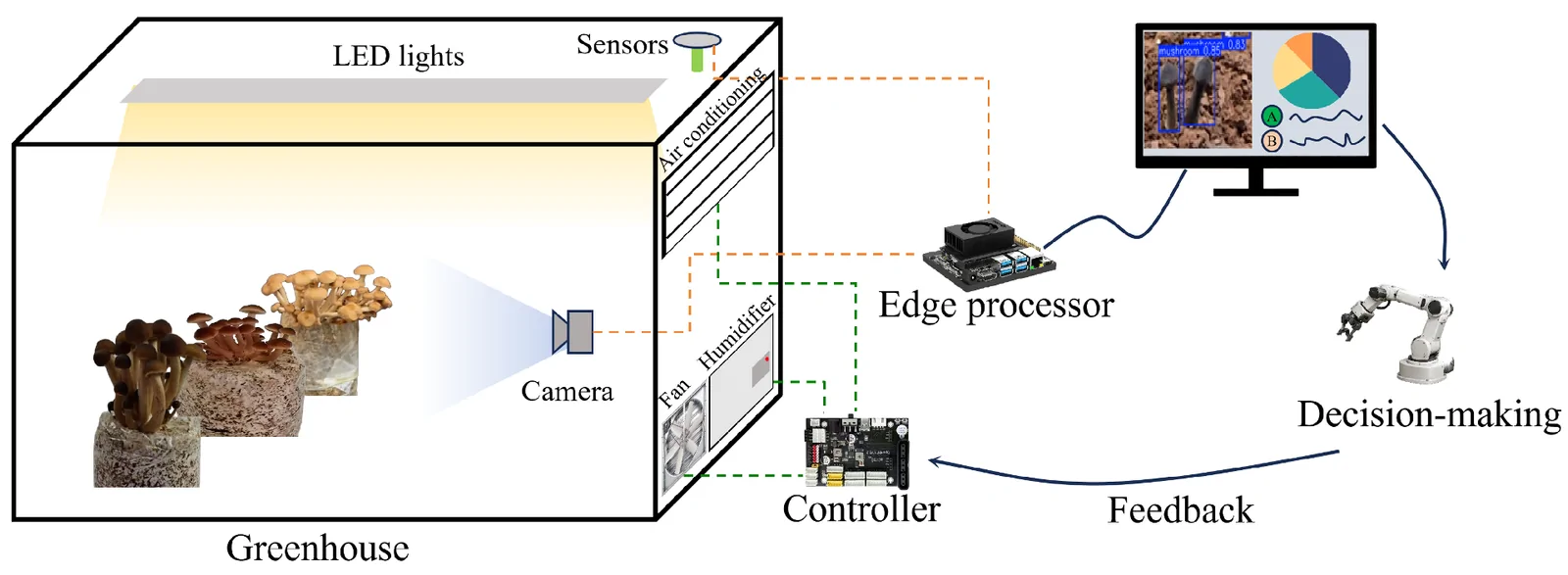
Edge-computing deployment architecture for RGB-based mushroom phenotyping, showing the data flow from fixed image acquisition and edge processing to production decision-making and closed-loop robotic and environmental control.

The second gap concerns deployability and system-level real-time performance. Production systems frequently operate on mobile, edge, embedded, or industrial-control platforms with limited computing capacity, memory, energy, and response time. Lightweight mobile recognition [99], conveyor-belt grading [100], and harvesting systems integrating edge computing and robotic control [103] demonstrate progress toward practical deployment. However, system efficiency is not determined by model FPS alone. Image acquisition frequency, motion blur, illumination changes, target overlap, device latency, conveyor speed, actuator response, and mechanical execution jointly determine throughput and reliability [100,104]. A representative edge-computing deployment architecture for mushroom production is illustrated in Figure 12. RGB images acquired by fixed cameras are transmitted to an edge-processing device for phenotypic analysis and production-decision generation, after which the resulting control commands can be forwarded to robotic or environmental-control systems, forming a perception–decision–execution closed loop.

A minimum FPS requirement should be derived from process kinematics rather than stated as a universal threshold. For a sorting line transporting *q* items per second and requiring *n* independent observations per item, perception must sustain at least $f_{\min}\geq nq$, while end-to-end latency must remain below the time available between detection and actuation. For a moving robotic arm, the requirement is determined by control-update frequency, permissible localization error, target speed, robot-cycle time, and actuator response. Published examples illustrate why a single threshold is misleading: CMCI-RTDETR reports 56.66 FPS for sorting-related grading [78], whereas a robotic harvesting system reports 6.52 FPS with a 73.9% picking success rate [30]. The former does not by itself prove end-to-end sorting throughput, and the latter can still be sufficient for a slow pick cycle. Deployment studies should therefore report edge-device FPS, p95 latency, frame loss, items processed per minute, and control-response time rather than only desktop or GPU inference speed.

The third gap is the conversion of visual outputs into production decisions and mechanical actions. Algorithmic metrics such as mAP, accuracy, F1-score, IoU, and measurement error do not directly represent harvesting success, product damage, grading throughput, equipment stability, or maintenance cost. Robotic harvesting studies show that detections and spatial estimates must be translated into target localization, picking-sequence planning, end-effector control, and low-damage operation [101,105]. Conveyor-belt grading similarly requires coordination among image acquisition, detection zones, belt speed, sorting rules, and actuators [100]. Perception errors may therefore propagate through downstream decision and control modules, particularly under dense growth or high-speed operation.

The fourth gap is economic feasibility for small and medium-scale producers. The relevant comparison should not be based on sensor price alone, but on total cost of ownership under defined accuracy, throughput, and operational-stability requirements. Exclusive use of monocular RGB avoids depth-camera, stereo, or multispectral hardware, reduces synchronization and multi-sensor calibration requirements, and simplifies system integration and maintenance [10,11]. These advantages create a substantial initial hardware cost advantage for monitoring, counting, size grading, and surface-quality pre-screening. However, this does not eliminate lighting, protective housing, edge computation, system integration, cleaning, recalibration, model maintenance, or downtime costs [14]. For safety- or success-critical robotic grasping, monocular two-dimensional or predicted depth may not meet the required spatial accuracy unless geometric priors, scale calibration, and uncertainty rejection maintain the necessary success rate [101,105]. Cost comparisons should therefore report the total cost of ownership, including hardware, installation, calibration, edge or cloud computing, maintenance, energy, retraining, and downtime, normalized as cost per 10,000 items or per cultivation cycle, together with accuracy, latency, and success rate. Only then can it be determined where the lower hardware investment of monocular RGB sufficiently compensates for reduced spatial precision and where higher-precision sensing remains necessary.

The fifth gap involves interpretability and standardization. For maturity assessment, quality grading, and safety-oriented recognition, a classification label alone may be insufficient for production decisions. Operators need to determine whether predictions rely on meaningful traits such as cap edges, texture, cracks, or color changes rather than irrelevant background information. Explainable mushroom-house monitoring has begun to visualize model attention regions [98], but definitions of maturity, quality grade, damage severity, and morphological traits remain inconsistent across studies. Unified annotation rules, phenotype definitions, evaluation metrics, uncertainty reporting, and risk-control mechanisms are therefore needed to make model outputs comparable, interpretable, and traceable.

Production-oriented progress consequently requires more than higher task-level accuracy. Models must generalize across species, batches, devices, and sites; operate stably under mobile, edge, conveyor, and robotic conditions; and be evaluated through complete perception–decision–execution pipelines. Deployment solutions must also demonstrate that they satisfy the required production cycle and achieve an acceptable total cost of ownership at the target accuracy and throughput. Only when visual phenotypes can be converted into reliable, interpretable, and executable production decisions can RGB-based mushroom phenotyping progress from isolated algorithm validation to sustained production use.

## 5. Future Perspectives

The findings synthesized in this review indicate that future progress in RGB image-based phenotypic analysis of edible mushrooms cannot be achieved solely by improving the accuracy of isolated recognition models. The current technical bottlenecks form a connected chain: incomplete target visibility affects individual separation; segmentation and boundary errors propagate into morphological measurements; two-dimensional projection limits the recovery of spatial traits; discrete quality and maturity labels simplify continuous biological variation; and task-level accuracy does not necessarily translate into production-level utility. Future research should therefore advance toward standardized data foundations, unified phenotype definitions, spatially consistent three-dimensional representation, temporal growth understanding, and closed-loop production validation.

Table 8 summarizes the major future directions derived from the methodological gaps identified in this review.

### 5.1. Standardized Multi-Task and Temporal Datasets

Current edible mushroom image datasets are commonly developed for a single task, such as object detection, instance segmentation, quality classification, species recognition, or maturity assessment. Although these datasets support task-specific model comparisons, they provide limited support for analyzing error propagation across detection, segmentation, measurement, spatial reconstruction, temporal tracking, and production decision-making. Their limited diversity in mushroom species, cultivation batches, imaging devices, viewpoints, illumination conditions, density levels, and occlusion patterns also restricts cross-scenario generalization.

Future datasets should be organized around complete cultivation units, fruiting-body populations, and growth trajectories rather than collections of independent images. The same image or image sequence should, where possible, provide mutually associated annotations, including bounding boxes, instance masks, cap and stipe regions, structural keypoints, visible and occluded regions, morphological measurements, quality attributes, species labels, maturity states, and temporal identities. For densely distributed fruiting bodies, annotations describing occlusion degree, adhesion relationships, instance visibility, and boundary confidence would be particularly valuable for evaluating individual separation and amodal recovery.

Multi-view and temporal data should become important components of future benchmarks. Multi-view images can support the evaluation of viewpoint consistency and three-dimensional reconstruction, while time-series images can support instance tracking, growth-curve estimation, maturity prediction, and harvest-window assessment. Acquisition metadata, including camera parameters, imaging distance, viewpoint, timestamp, cultivation batch, mushroom-house location, and environmental records, should also be retained.

For a subset of the data, manual measurements, calibrated scale references, depth maps, laser scans, or reconstructed point clouds may be collected as reference information, even when the evaluated model uses only RGB images as input. Such reference data would make it possible to distinguish algorithmic improvements from actual improvements in phenotypic accuracy. Dataset partitions should also be defined according to cultivation site, production batch, imaging device, or acquisition period rather than relying exclusively on random image splitting, which may overestimate model generalization when visually similar samples occur in both training and test sets.

Training and validation/test partitions must also be separated at the level of farm or mushroom house, strain, and seasonal lighting regime, with results reported under leave-one-farm-out, leave-one-strain-out, and leave-one-lighting-regime-out protocols. Image-level random splitting may be retained only as a diagnostic baseline and cannot be interpreted as evidence of generalization. Seasonal lighting regimes should record the combination of natural and artificial light, photoperiod, and light-intensity conditions; stable performance across these independent domains is necessary before a model can be considered deployable across production conditions.

### 5.2. Unified Phenotype Definitions and Reliability Evaluation

A central limitation of current research is the absence of consistent definitions for visible and spatial phenotypic indicators. Cap diameter may be calculated from a bounding rectangle, fitted ellipse, maximum Feret diameter, visible contour, or recovered amodal mask. Similarly, stipe length may refer to a straight-line distance, skeleton length, centerline length, or projected height. Maturity, damage, freshness, browning, and commodity grade are also frequently defined according to study-specific experience or production standards. Consequently, apparently identical indicators may represent different biological or geometric properties across studies.

Future research should distinguish at least three levels of phenotype output. The first comprises directly observed phenotypes, such as visible projected area, color, texture, and contour. The second comprises model-inferred phenotypes, including completed occluded shapes, relative depth, absolute dimensions, volume, and curvature. The third comprises production-oriented indicators derived from these phenotypes, such as harvestable proportion, commodity grade, harvest priority, and yield estimates. Clearly identifying whether an indicator is directly observed, geometrically estimated, or model-predicted would improve the interpretability and comparability of reported results.

Evaluation should accordingly move beyond mAP, IoU, Dice, classification accuracy, and FPS. Detection studies should report counting error, duplicate detection, missed detection, and localization errors relevant to harvesting or yield estimation. Segmentation studies should examine how mask errors affect cap diameter, area, perimeter, roundness, stipe length, and other structural traits. Spatial estimation studies should report scale error, depth-order consistency, orientation error, volume error, and reconstruction completeness. Quality assessment should consider ordinal agreement, continuous defect severity, and cross-batch consistency rather than only discrete classification accuracy.

Reliability assessment should also connect the direction of errors to production consequences. When the positive class denotes the optimal harvest window, a false positive can lead to premature harvesting, reduced fresh weight and grade, and additional inspection and labor, whereas a false negative can result in overmaturity, weight loss, or rejection; in grading, undergrading sacrifices sales revenue, while overgrading causes rework and returns. These consequences should be converted into expected net-margin loss per square meter per production cycle using error prevalence, fruit density, unit fresh weight, grade-price differentials, and additional labor or energy costs, enabling the reporting of a break-even error rate and the use of cost-sensitive thresholds.

Species identification additionally requires open-set and risk-sensitive evaluation because an unknown or poisonous species should not be treated in the same manner as an ordinary classification error. Confidence calibration, uncertainty estimation, rejection of unfamiliar samples, and analysis of whether predictions depend on biologically meaningful structures or background correlations should therefore receive greater attention. These evaluation principles would shift the focus from whether a model produces the correct label on a fixed dataset to whether its phenotypic output remains reliable, interpretable, and safe under changing conditions.

### 5.3. Spatially Consistent 3D Phenotyping

The newly emerging studies on RGB-based depth estimation and three-dimensional reconstruction indicate an important extension of edible mushroom phenotyping beyond two-dimensional projected traits. However, multi-view reconstruction, monocular depth prediction, and geometry-assisted approximation should not be treated as equivalent technical routes. Multi-view reconstruction can recover relatively complete point clouds and metrically meaningful spatial traits, but requires coordinated acquisition from multiple viewpoints, sufficient surface texture, and substantial post-processing. Monocular depth prediction is easier to deploy, but commonly produces relative depth with scale ambiguity. Geometry-assisted methods can improve size estimation or recover partially occluded structures, but their results depend strongly on scene-specific assumptions, camera calibration, and anatomical priors.

Future research should establish clearer task definitions and evaluation protocols for these three routes. Multi-view reconstruction should be assessed in terms of point-cloud completeness, surface accuracy, acquisition efficiency, and robustness to weak texture and occlusion. Monocular depth prediction should explicitly distinguish relative depth ordering from metric depth recovery and should report performance across species, viewpoints, illumination conditions, and cultivation structures. Geometry-assisted inference should report the validity range of its structural assumptions and the sensitivity of estimated traits to changes in camera pose and fruiting-body orientation.

The objective of RGB-based spatial phenotyping should not be to replace every two-dimensional method with a complex three-dimensional pipeline. Instead, future systems should determine which production tasks genuinely require spatial information. Two-dimensional contours may be sufficient for visible defect detection, projected-area monitoring, or simple grading, whereas volume estimation, stipe orientation, occlusion interpretation, collision-free harvesting, and complete morphology reconstruction require more reliable spatial representations. Hybrid frameworks could therefore use efficient two-dimensional analysis for routine monitoring and activate multi-view reconstruction, depth prediction, or geometric reasoning only when spatial information is required.

Paired RGB images and reference point clouds or depth measurements would facilitate the development of scale-aware reconstruction and depth models while preserving RGB images as the primary operational input. Cross-view consistency, temporal consistency, anatomical plausibility, and uncertainty in invisible regions should also be incorporated into future spatial models. These developments would promote a transition from approximate two-dimensional measurements toward spatially consistent phenotypic representations without losing the low-cost deployment advantages of RGB imaging.

### 5.4. Temporal Growth Modeling and Decision Prediction

Single-frame RGB images describe the visible state of fruiting bodies at a specific moment but cannot fully represent continuous development from primordium emergence to enlargement, maturity, and harvesting. Although recent studies have introduced time-lapse segmentation, instance tracking, maturity-time prediction, and future-image generation, temporal analysis remains affected by small early-stage targets, rapid morphological change, merging and splitting of clusters, occlusion, illumination variation, newly emerging individuals, and partial harvesting.

Future temporal models should establish persistent associations for individual fruiting bodies or biologically meaningful clusters across successive observations. The appropriate tracking unit may differ among mushroom species and cultivation modes; therefore, instance-level and cluster-level tracking should be evaluated separately. Models should also explicitly handle the appearance of new fruiting bodies, disappearance caused by harvesting, temporary loss caused by occlusion, and changes in visible structure caused by cap expansion.

Temporal understanding should integrate changes in size, projected area, spatial depth, cap opening, color, texture, population coverage, and growth rate rather than relying exclusively on discrete stage labels. Environmental records may be incorporated as contextual variables to explain growth differences and improve prediction, while image-derived phenotypes should remain the primary evidence for visible developmental changes. Future outputs should progress from statements such as “the mushroom is mature” toward predictions of when an individual or cultivation batch will enter the optimal harvest window.

Prediction uncertainty is also important because growth rates vary across individuals, batches, and environmental conditions. Harvest-time models should therefore provide confidence intervals or risk levels and should be validated across independent cultivation cycles. By combining temporal instance association, growth-curve modeling, spatial information, and uncertainty-aware prediction, RGB imaging could support not only retrospective growth description but also forward-looking harvest scheduling, yield forecasting, and abnormal-growth warning.

### 5.5. Closed-Loop Production Systems

The ultimate value of image-based phenotypic analysis lies not in producing bounding boxes, masks, depth maps, or classification labels, but in converting these outputs into reliable production actions. Future studies should therefore evaluate complete perception–decision–execution pipelines rather than isolated visual modules. Detection and spatial estimation should be linked to harvesting localization and collision avoidance; structural and quality phenotypes should be linked to grading and sorting rules; maturity prediction should support harvest scheduling and labor or robot allocation; and abnormal growth detection should support production warnings and environmental management.

System-level real-time performance cannot be represented by model FPS alone. It also depends on image acquisition frequency, image transmission, preprocessing, target motion, control latency, actuator response, conveyor-belt speed, harvesting sequence, and mechanical recovery after failed operations. Errors introduced during perception may be amplified during downstream decision and execution. Production validation should therefore trace how detection, segmentation, measurement, depth, and classification errors influence final harvesting success, product damage, grading consistency, throughput, and equipment stability.

Future evaluation protocols should include long-term operation across cultivation batches and should report harvesting success rate, damage rate, grading throughput, missed-object rate, actuator response time, system downtime, energy consumption, maintenance requirements, and economic benefits. Cross-device recalibration, model drift detection, failure recovery, and human intervention mechanisms should also be considered. For quality grading, maturity assessment, and safety-oriented species recognition, interpretable evidence and uncertainty warnings should remain available to production personnel rather than being hidden within an automated decision pipeline.

Closed-loop validation should compare RGB-assisted management with conventional or sensor-rich management in paired blocks within the same facility and cover at least one complete production cycle. In addition to task-level metrics, studies should report marketable yield, Grade A proportion, labor, water use, energy use, and net margin per square meter per cycle. Improvements in net margin should be decomposed into reduced decision errors, resource and labor savings, hardware and maintenance costs, and downtime, thereby identifying the break-even error rate and defining where monocular RGB can replace or complement more expensive sensing systems.

Through these developments, future research can move from low-cost RGB image recognition toward standardized, spatially informed, temporally predictive, and production-deployable phenotyping. Connecting visible phenotype extraction, three-dimensional spatial representation, temporal growth modeling, reliability evaluation, and closed-loop decision-making will be essential for transforming monocular RGB imaging from an experimental analytical tool into a dependable component of intelligent edible mushroom production.

## 6. Conclusions

This review examined phenotypic analysis of edible mushroom fruiting bodies using monocular RGB images from a problem-oriented perspective. Rather than organizing the literature primarily by model architecture, it established an analytical framework linking visible phenotypes, visual tasks, methodological bottlenecks, and production applications. The reviewed studies were organized around six major research problems: small-object localization and individual separation; structural segmentation, occlusion recovery, and morphological measurement; RGB-based three-dimensional reconstruction and spatial estimation; appearance-based quality assessment and species identification; temporal growth analysis and maturity assessment; and production-oriented deployment.

Considerable progress has been achieved in fruiting-body detection, instance segmentation, structural measurement, appearance recognition, growth monitoring, and automated production. Multi-scale feature fusion and contextual modeling have improved the detection of small and densely distributed targets, while instance segmentation and amodal recovery have extended analysis from approximate localization toward individual separation and complete-shape inference. Structural segmentation, edge detection, keypoint estimation, and geometric modeling have enabled quantitative extraction of cap, stipe, and whole-fruiting-body traits. Recent studies have also expanded conventional two-dimensional analysis toward monocular depth prediction, geometry-assisted inference, multi-view RGB reconstruction, temporal tracking, and growth prediction.

Nevertheless, the reliability and production applicability of current methods remain limited. Two-dimensional projections cannot fully represent physical size, depth, orientation, curvature, volume, or occluded structures, while monocular depth prediction and multi-view reconstruction involve scale ambiguity or additional acquisition complexity. Phenotypic indicators, maturity stages, and quality grades are also defined inconsistently across studies, reducing comparability and reproducibility. Appearance recognition remains sensitive to illumination, viewpoint, background, device, and regional variation, whereas temporal models are affected by occlusion, newly emerging individuals, partial harvesting, and accumulated prediction errors. In addition, many studies continue to emphasize task-level metrics on limited datasets rather than cross-batch generalization, phenotype reliability, and long-term production performance.

Future progress requires standardized multi-task and temporal datasets, unified phenotype definitions, uncertainty-aware evaluation, spatially consistent analysis, temporal growth modeling, and production-level validation. Linking visible phenotype extraction, spatial estimation, growth prediction, and perception–decision–execution pipelines will be essential for transforming RGB-based mushroom phenotyping from isolated experimental models into reliable tools for intelligent production.

Ultimately, the strategic value of monocular RGB imaging lies not only in technical performance but also in commercial accessibility: its lower capital intensity and maintenance burden offer small and medium-scale producers a comparatively affordable route to automation. Once validated in closed-loop protected cultivation, reliable RGB phenotyping can improve harvest timing, grading, and environmental management, reducing unnecessary water, energy, and labor consumption while increasing marketable yield and net margin per square meter. Its broader significance therefore lies in combining low-cost visual intelligence with commercial inclusiveness and resource-efficient protected agriculture.

## Figures and Tables

**Table 1:** Main visible phenotype categories of edible mushroom fruiting bodies based on monocular RGB images.

Phenotype Category	Main Concern	Representative Indicators	Applications and Major Limitations
Morphological and structural phenotypes	Size, shape, number, location, and spatial organization	Cap diameter, area, perimeter, roundness, stipe traits, number, density, spacing, and coverage	Size grading, yield estimation, and harvesting localization; limited by small targets, occlusion, adhesion, viewpoint, and scale calibration
Appearance quality phenotypes	Commodity condition and visible surface defects	Color, brightness, texture, cracks, spots, decay, contamination, deformity, browning, and damage	Quality grading, defect detection, and postharvest sorting; limited by illumination variation, color drift, subtle defects, developmental appearance changes and inter-batch distribution shifts, and inconsistent standards
Growth- and maturity-related phenotypes	Developmental state, growth dynamics, and harvest readiness	Number change, area growth, cap expansion, opening degree, coverage, and mature proportion	Growth monitoring and harvest prediction; limited by tracking instability, stage ambiguity, occlusion, and batch differences

**Table 2:** Representative studies on small-object localization and individual separation in dense or complex mushroom scenes.

Study	Task and Method	Contributions, Reported Performance, and Robustness Limitations (Domain Shift, Occlusion/Adhesion, Illumination/Viewpoint, Measurement Validity, Deployment)
Wei et al. [56]	Detection in cultivation-stick scenes; Recursive YOLOv5 with ASPP and DIoU-NMS	Contribution: High-resolution small-target localization. Performance: 98% recognition, +12.87 points over YOLOv5X. Limitation: Doubled parameters; no counting error, latency, severe-occlusion, or cross-farm validation.
Chen et al. [59]	*Agaricus bisporus* detection, counting, and size measurement; YOLOv5s-CBAM with Mosaic augmentation	Contribution: Detection, counting, center localization, and approximate sizing. Performance: 98% recognition; 18 ms/image; 0.40% center error; 1.08% diameter error. Limitation: No counting MAE or adhesion/domain-shift validation.
Zhao et al. [60]	*O. raphanipes* detection; YOLOv5s with RFBSE, DCSPP, RFP, and Focal-EIoU	Contribution: Robust detection under weak light and similar backgrounds. Performance: mAP 90.8%, precision 86.5%, recall 84.8% (+2.7/+3.8/+3.9 points). Limitation: No counting error, edge latency, or cross-scene validation.
Ye et al. [55]	Small fruiting-body detection; MSH-YOLOv8 with scale reconstruction and feature fusion	Contribution: Improved tiny-target representation. Performance: ${\mathrm{AP}}_{50}$ 98.49%, ${\mathrm{AP}}_{50:95}$ 75.29%, small-object AP 39.73%. Limitation: Weak strict-localization; occlusion and cross-dataset shift remain untested.
Xu et al. [61]	Shiitake instance segmentation; YOLOv5seg-BotNet with BoTNet, PANet, and Varifocal Loss	Contribution: Pixel-level instances for counting and grading. Performance: Precision 97.58%, recall 95.74%, Mask AP 95.90%, F1 96.65%, 32.86 FPS. Limitation: Adhesion correspondence, edge deployment, and cross-device shift remain unverified.
Yang et al. [62]	Dense instance and contour segmentation; PR-SOLOv2 with PointRend and contour reconstruction	Contribution: Boundary and center recovery in dense clusters. Performance: 93.04% segmentation accuracy, 98.13% success, 0.3% center error. Limitation: Adhesion, illumination, viewpoint, scale, and edge FPS remain challenging.
Wang et al. [57]	Log-cultivated shiitake detection; RT-DETR with FasterNet, SFFN, SPDConv, CSPOmniKernel, and EIoU	Contribution: Lightweight detection for small, dense, low-light targets. Performance: Accuracy 95.8%, recall 93.1%, mAP 95.3%, 19.1 M parameters, 53.6 GFLOPs. Limitation: Thresholds/splits unclear; no edge latency or domain-shift test.
Tan et al. [58]	Dense brown mushroom localization; YOLOv7 with ELAN-PS, AFPN, and MDIoU	Contribution: Improved localization in dense, occluded populations. Performance: +15.5% speed, +6.4% accuracy, +6.9 points mAP@0.5. Limitation: Absolute mAP/FPS absent; connected regions and cross-device shift unresolved.

**Table 3:** Representative studies on structural segmentation, occlusion recovery, and morphological measurement.

Study	Task and Method	Contributions, Reported Performance, and Robustness Limitations (Domain Shift, Occlusion/Adhesion, Illumination/Viewpoint, Measurement Validity, Deployment)
Wang et al. [68]	Fresh *Agaricus bisporus* diameter measurement; thresholding, double watershed, Canny edges, and morphology	Contribution: Shadow/stipe removal for diameter estimation. Performance: Mean error 1.657%; range 0.05–4.32% (20 samples). Limitation: Pixel-calibrated; reflections, incomplete contours, and cross-device shift remain limiting.
Zhu and Zhang [69]	High-throughput morphology of *Flammulina filiformis*; binarization, contour extraction, and cap-attachment detection	Contribution: Standardized batch extraction of cap/stipe morphology and color. Performance: RMSPE/MAPE generally (<0.05); stipe-width error (<0.06). Limitation: Requires detached/scanned samples; field throughput and cross-batch shift unverified.
Wei et al. [54]	Complete cap recovery under occlusion; synthetic occlusion and amodal instance segmentation	Contribution: Low-cost synthetic occlusion for complete cap recovery. Performance: ${\mathrm{AP}}_{50:95}$ 82–96%; length/width $R^{2}=0.95/0.98$. Limitation: Synthetic-to-real shift, millimeter error, and extreme occlusion unverified.
Yin et al. [53]	Occluded *O. raphanipes* cap measurement; OR-ORCNN, amodal mask, and pinhole camera model	Contribution: Geometry-assisted recovery of complete cap dimensions. Performance: ${\mathrm{AP}}_{50}$ 91.656%, mAP 76.046%, MAE 0.93 mm, RMSE 1.29 mm. Limitation: mAP falls to 68.603% above 30% occlusion; calibration and cross-farm shift remain limiting.
Tao et al. [8]	Shiitake cap geometry and selective harvesting evaluation; ReYOLO-MSM with rotated detection and stick–mushroom geometry	Contribution: Monocular cap sizing and harvest-batch evaluation. Performance: 99.3% accuracy; 98.9% mAP; 7.1 ms/frame; 4.55 mm maximum error; 2.18% mean error. Limitation: Assumes stable stick geometry/orientation; domain shift and cycle time unverified.
Zhao et al. [24]	Shiitake cap trait extraction; KL-DexiNed edge detection and OpenCV calculation	Contribution: Cap trait extraction and weight prediction. Performance: OIS 93.5%, ODS 96.3%, AP 97.1%, prediction $R^{2}=0.97$, RMSE 0.049. Limitation: Unit/normalization unclear; inclination, thickness, and cross-device shift remain problematic.
Choi et al. [70]	Cap and stipe segmentation and measurement; YOLOv8-seg and YOLOv11-seg with caliper validation	Contribution: Matched-segmentation benchmark with caliper validation. Performance: Δ${\mathrm{mAP}}_{50:95}<0.01$; YOLOv11 uses fewer parameters/FLOPs. Limitation: Trait-level MAE/RMSE missing; occlusion and domain shift remain limited.
Zhao et al. [67]	Shiitake stipe trait measurement; YOLOv11n, EfficientSAM, OpenCV, and centerline prediction	Contribution: Curved stipe measurement via segmentation and centerline prediction. Performance: AP 97.2%, recall 96.1%, 22.09 ms; $R^{2}$ = 0.978–0.992; RMSE 0.023–0.043. Limitation: Curvature, occlusion, cross-species shift, and edge latency remain.

**Table 4:** Representative studies on three-dimensional morphological reconstruction and spatial information estimation from RGB images.

Study	Task and Method	Contributions, Reported Performance, and Robustness Limitations (Domain Shift, Occlusion/Adhesion, Illumination/Viewpoint, Measurement Validity, Deployment)
Xu et al. [73]	Shiitake 3D reconstruction, trait extraction, and yield estimation; smartphone multi-view RGB imaging; SfM; MVS; CP-PointNet++	Contribution: Smartphone-to-point-cloud pipeline for 3D traits and yield. Performance: Segmentation OA 97.45%; cap diameter $R^{2}>0.8$; cap height RMSE 3.26 mm; stipe height RMSE 4.98 mm; yield $R^{2}=0.91$. Limitation: Multi-view/texture dependence; weak texture, occlusion, viewpoint shift, and real-time deployment remain limiting.
Wang et al. [74]	Oyster mushroom segmentation and depth prediction; ConvNeXt encoder, FPN, and lightweight segmentation/depth heads	Contribution: RGB-only joint segmentation and depth estimation. Performance: IoU 0.935; depth RMSE 26.1 mm (705 samples). Limitation: Single-scenario validation; cross-farm, substrate, background, and illumination shift unresolved.
Yin et al. [53]	Occluded *Oudemansiella raphanipes* cap measurement; amodal mask and pinhole camera geometry	Contribution: Geometry-assisted recovery of complete cap dimensions. Performance: ${\mathrm{AP}}_{50}$ 91.656%, mAP 76.046%, MAE 0.93 mm, RMSE 1.29 mm. Limitation: mAP falls to 68.603% above 30% occlusion; calibration and embedded cost remain limiting.
Tao et al. [8]	Shiitake cap geometry and selective harvesting evaluation; monocular detection and stick–mushroom growth geometry	Contribution: Low-cost monocular sizing from stick–mushroom geometry. Performance: Maximum error of 4.55 mm; 2.18% mean error. Limitation: Assumes stable geometry/orientation; occlusion, camera movement, and humidity calibration remain untested.
Wei et al. [54]	Complete cap-shape recovery under occlusion; synthetic occlusion and amodal instance segmentation	Contribution: Low-cost synthetic occlusion for complete-shape recovery. Performance: ${\mathrm{AP}}_{50:95}$ 82–96%; length/width $R^{2}=0.95/0.98$. Limitation: 2D masks do not measure volume, curvature, or absolute depth; real-world shift remains unverified.

**Table 5:** Representative studies on appearance-based quality assessment and species identification.

Study	Application, Task, and Method	Contributions, Reported Performance, and Robustness Limitations (Domain Shift, Occlusion/Adhesion, Illumination/Viewpoint, Measurement Validity, Deployment)
Quality; Wang et al. [76]	Dried shiitake grading; OOA-Otsu and D-VGG	Contribution: Automated six-grade recognition. Performance: 96.21% accuracy; 46.77 ms/image. Limitation: Threshold-specific; cross-batch/device shift unvalidated.
Quality; Wang et al. [78]	Shiitake grading and defect detection; CMCI-RTDETR	Contribution: Lightweight grading and small-defect localization. Performance: mAP@0.5 84.66%, mAP@0.5:0.95 71.54%, 12.5 M parameters, 56.66 FPS. Limitation: Device/resolution unspecified; defect thresholds and domain-shift tests incomplete.
Quality; Li et al. [79]	Shiitake drying-quality measurement; online segmentation and pixel statistics	Contribution: Online wrinkling and shrinkage measurement. Performance: Shrinkage error 0.02–0.13; wrinkled-area error 6–7%. Limitation: Processing-specific; fresh-product and cross-device transfer unverified.
Quality; Lv et al. [38]	*Stropharia rugosoannulata* grading and sorting; YOLOv8-seg, OpenCV, SegGrade, and air-blown sorting	Contribution: Segmentation-to-sorting pipeline. Performance: 99.5% segmentation; 94.67% SegGrade; 80.66% system accuracy. Limitation: Large system-level drop; integrity, actuator latency, humidity, and domain shift unreported.
Species; Ketwongsa et al. [86]	Thai edible–poisonous classification; improved AlexNet	Contribution: Regional edible–poisonous classification. Performance: CNN 98.50%; R-CNN 95.50%. Limitation: Five species only; open-set rejection, cross-region shift, and toxicity false negatives unresolved.
Species; Lim et al. [90]	Malaysian species recognition; regional dataset with ViT and ResNet	Contribution: Regional comparison of transformer and CNN classifiers. Performance: ViT-L/16 90.47% accuracy. Limitation: No macro-F1, unknown-category rejection, or geographic transfer.
Species; Ahmed and Kou [91]	Wild mushroom recognition; ResNet and region-based CNNs	Contribution: Fine-grained species discrimination. Limitation: Full text unavailable; metrics, confusion structure, and domain shift require verification.
Species; Zhao et al. [93]	Fine-grained edible mushroom classification; AWPF-ResNet18	Contribution: Stronger multi-scale texture and stipe features. Performance: Accuracy/precision/F1/recall gains of 2.5/7.5/5/2 points. Limitation: Absolute metrics, cross-species tests, occlusion, and edge cost needed.

**Table 6:** Dedicated summary of temporal growth and maturity assessment.

Study	Temporal Task and Method	Contributions, Reported Performance, and Robustness Limitations (Domain Shift, Occlusion/Adhesion, Illumination/Viewpoint, Temporal Consistency, Deployment)
Charisis et al. [26]	Time-lapse segmentation, tracking, and area monitoring; Mask R-CNN with ConvNeXt/Swin and mask-based tracking	Contribution: Time-lapse segmentation, tracking, and growth curves. Performance: mAP 0.876; F1 0.867; MOTA 0.967. Limitation: Illumination, occlusion, partial harvest, and manual thresholds affect consistency.
Xu et al. [95]	Fine-grained maturity detection; YOLOv8n-CFS with SegNeXt attention, FDPN, and CSFCN	Contribution: Improved subtle maturity-state discrimination. Performance: ${\mathrm{mAP}}_{50}$ 97.34%; ${\mathrm{mAP}}_{95}$ 84.5%. Limitation: Single-frame; no time or environmental feedback; cross-batch shift uncertain.
Yang et al. [97]	On-site viable-primordia counting; RSL-YOLOv8m-seg, CLAHE, and convex hull fitting	Contribution: Viable-primordia segmentation and counting. Performance: mAP@0.5 68.1%; recall 48.5%; TPR 89.2%; FNR reduced by 19.1 points. Limitation: Low mAP/recall; irregular morphology and annotation consistency.
Nuwayhid et al. [64]	Multi-view growth tracking; Mask R-CNN with Swin and relative-area curves	Contribution: Multi-view instance tracking and growth curves. Performance: Front/side/top mAP 0.82/0.86/0.95. Limitation: Early targets and low light cause misses; pixel area lacks physical calibration.
Moysiadis et al. [94]	Growth monitoring and harvest-day indication; YOLOv5 for stage detection and Detectron2 for monitoring	Contribution: Stage detection linked to growth and harvest timing. Performance: F1 76.5%; final-stage accuracy 70%; growth 5.22 days. Limitation: Modest accuracy; no environmental time-series or cross-batch validation.
Fan et al. [45]	Maturity-time prediction; YOLO-P, YOLOv12, Swin Transformer v2, logistic fitting, and recursive Gaussian fusion	Contribution: Detection plus continuous maturity-time prediction. Performance: mAP@50 97.2%; maturity MAE 0.42 days; R=0.86. Limitation: Low light and batch variation remain; no temperature/humidity input.
Wang et al. [44]	Growth-stage detection and segmentation; GSP-RTMDet with SPPF-SAM, CSP-SGE, and CARAFE	Contribution: Lightweight stage detection and segmentation. Performance: bbox mAP 96.40%; segm mAP 93.70%. Limitation: No FPS/p95 latency; overlap and cross-farm shift unresolved.
Ahmed et al. [98]	CCTV-based maturity monitoring; attention-enhanced ShuffleNetV2 with Grad-CAM, EigenCAM, and LIME	Contribution: Lightweight, explainable CCTV monitoring. Performance: 99.70% accuracy; 1.3 M parameters; 2.77 FPS; 48 ms latency. Limitation: Low-resolution/video degradation; adequate for minute-level monitoring, not high-speed sorting.
Xu et al. [43]	Long-term growth prediction; VMRNN-DMSA with Mamba-RNN, skip connections, and spatial attention	Contribution: Long-term growth-image prediction. Performance: MSE 39.4255; SSIM 0.8579; PSNR 22.0774. Limitation: Image metrics do not measure maturity-time error; drift and environmental feedback unresolved.

**Table 7:** Dedicated summary of production-oriented applications, hardware, throughput, latency, and environmental constraints.

Study	Application, Hardware, and System	Production Contribution, Deployment Performance, and Robustness Limitations (Domain Shift, Occlusion/Adhesion, Throughput, End-to-End Latency, Humidity)
Zhu and Zhang [69]	High-throughput breeding phenotyping; standardized RGB acquisition and automated trait extraction	**Contribution:** Standardized batch phenotyping. **Performance:** RMSPE/MAPE generally (<0.05). **Limitation:** Laboratory sample preparation; no line FPS, latency, or humidity protection.
Ahmed et al. [98]	Continuous mushroom-house monitoring; CCTV, attention-enhanced ShuffleNetV2, and explainable AI	Contribution: Low-cost, interpretable CCTV monitoring. Performance: 99.70% accuracy; 1.3 M parameters; 2.77 FPS; 48 ms latency. Limitation: No p95 latency, frame loss, uptime, or condensation control.
Peng et al. [99]	Mobile species recognition; MobileNetV3 on PC, Android, and Raspberry Pi 4B	Contribution: Multi-platform mobile recognition. Performance: 98.13%/97.98% validation/test; Android 50 ms; (<200) MB. Limitation: No edge FPS, power, humidity durability, or open-set testing.
Wei et al. [102]	Toxic-mushroom detection; PM-YOLO with StarBlock-CAA, CSFCN, and knowledge distillation	Contribution: Lightweight detection in complex habitats. Performance: mAP@0.5 92.64%; 3.5 M parameters; 10-fold variation (<2%). Limitation: RTX 3090 only; edge FPS, latency, maintenance, and geographic shift unresolved.
Shui et al. [100]	Conveyor-belt size grading; top camera, conveyor, lightweight YOLOv5, zonal detection, and online sorting	Contribution: Lightweight detection linked to online sorting. Performance: mAP@0.5 97.1%; 1066.67 mushrooms/min; 97.87% accuracy at 46.83–130.17 mm/s. Limitation: No absolute FPS, p95 latency, actuator response, or humidity protection.
Rong et al. [101]	Greenhouse robotic harvesting; Intel RealSense D435i, improved SSD, and soft gripper	Contribution: Field-tested perception-to-grasping system. Performance: 95% recognition; 86.8% harvesting success; 8.85 s/mushroom. Limitation: No damage, p95 cycle, positioning error, or humidity data.
Ma et al. [103]	Intelligent *Agaricus bisporus* harvesting; FES-YOLOv5s, NVIDIA Jetson Orin Nano, gantry arm, and flexible gripper	Contribution: Dense detection plus low-damage robotic harvesting. Performance: 96.72% recognition; 94.95% success; 2.67% damage; 0.91 mm RMSE. Limitation: No Jetson FPS, p95 latency, power, or humidity control.
Zhong et al. [104]	Autonomous dense-cluster harvesting; Improved YOLOv5s, Intel RealSense D435i, Jetson TX2, DBSCAN planning, and telescopic end-effector	Contribution: Overlap-aware detection, planning, and telescopic harvesting. Performance: AP 92.9%/96.7%/94.3%; localization error 1.34%/1.52%/2.36%; 94.1% success; 4.23 s/mushroom. Limitation: Occlusion errors, domain shift, p95 latency, and condensation unresolved.
Ji et al. [105]	Mushroom picking-platform development; depth camera, industrial PC, truss robot, constant-force gripper, and LabVIEW control	Contribution: Depth-based picking platform with constant-force grasping. Performance: 93.20% recognition; 96.34% success; 2.21% damage; 8.59 mushrooms/min. Limitation: Edge reachability, adhesion damage, p95 latency, and humidity protection unresolved.
Li et al. [30]	Robotic harvesting; Enhanced YOLOv11s, structured-light camera, ROS2, and robotic arm	Contribution: Detection-to-robot harvesting pipeline. Performance: mAP@0.50 96.7%; mAP@0.50:0.95 81.2%; 73.9% success; 6–14 mm end-to-end error. Limitation: No p95 latency/humidity protection; paper reports conflicting 142 and 6.52 FPS.

**Table 8:** Priority directions for future RGB image-based phenotypic analysis of edible mushrooms.

Future Direction	Current Limitation	Priority Actions
Standardized data foundations	Existing datasets are usually task-specific, species-specific, and collected under limited conditions.	Construct multi-task, multi-view, and temporal datasets with hierarchical annotations, acquisition metadata, reference measurements, and cross-site evaluation splits.
Unified phenotype definitions and reliability evaluation	Phenotypic indicators and grading criteria are inconsistently defined, while algorithmic metrics do not directly represent phenotype accuracy.	Standardize trait definitions, distinguish observed and inferred phenotypes, quantify uncertainty, and connect model metrics with measurement and decision errors.
Spatial and three-dimensional phenotyping	Two-dimensional projections cannot fully represent volume, orientation, curvature, occluded structures, or absolute spatial relationships.	Develop and compare multi-view reconstruction, monocular depth prediction, and geometry-assisted inference using scale calibration and reference spatial data.
Temporal growth understanding	Single-frame recognition cannot describe individual growth trajectories, developmental transitions, or future harvest readiness.	Strengthen cross-time tracking, growth-curve modeling, maturity-window prediction, and multimodal temporal association.
Closed-loop production validation	Most studies remain focused on offline algorithm evaluation rather than continuous production operation.	Evaluate complete perception–decision–execution pipelines using harvesting, grading, throughput, stability, and economic indicators.

## Data Availability

The dataset supporting the descriptive and thematic analyses in this review, including the bibliographic information and thematic classification of the 76 included studies, is publicly available in the GitHub repository at https://github.com/zhaowei9181/Overview-of-Mushrooms (accessed on 15 September 2026).
